# METTL1 promotes hepatocarcinogenesis via m^7^G tRNA modification‐dependent translation control

**DOI:** 10.1002/ctm2.661

**Published:** 2021-12-12

**Authors:** Zhihang Chen, Wanjie Zhu, Shenghua Zhu, Kaiyu Sun, Junbin Liao, Haining Liu, Zihao Dai, Hui Han, Xuxin Ren, Qingxia Yang, Siyi Zheng, Baogang Peng, Sui Peng, Ming Kuang, Shuibin Lin

**Affiliations:** ^1^ Department of Liver Surgery The First Affiliated Hospital Sun Yat‐sen University Guangzhou China; ^2^ Department of Gastroenterology and Hepatology The First Affiliated Hospital Sun Yat‐sen University Guangzhou China; ^3^ Department of Gastrointestinal Surgery The First Affiliated Hospital Sun Yat‐sen University Guangzhou China; ^4^ Center for Translational Medicine The First Affiliated Hospital Sun Yat‐sen University Guangzhou China; ^5^ Institute of Precision Medicine The First Affiliated Hospital Sun Yat‐sen University Guangzhou China; ^6^ Cancer Center, The First Affiliated Hospital Sun Yat‐sen University Guangzhou China; ^7^ State Key Laboratory of Oncology in South China Sun Yat‐sen University Cancer Center Guangzhou China

**Keywords:** hepatocellular carcinoma, N7‐methylguanosine, translation control, tRNA modifications, tumour progression

## Abstract

**Background:**

N^7^‐methylguanosine (m^7^G) modification is one of the most common transfer RNA (tRNA) modifications in humans. The precise function and molecular mechanism of m^7^G tRNA modification in hepatocellular carcinoma (HCC) remain poorly understood.

**Methods:**

The prognostic value and expression level of m^7^G tRNA methyltransferase complex components methyltransferase‐like protein‐1 (METTL1) and WD repeat domain 4 (WDR4) in HCC were evaluated using clinical samples and TCGA data. The biological functions and mechanisms of m^7^G tRNA modification in HCC progression were studied in vitro and in vivo using cell culture, xenograft model, knockin and knockout mouse models. The m^7^G reduction and cleavage sequencing (TRAC‐seq), polysome profiling and polyribosome‐associated mRNA sequencing methods were used to study the levels of m^7^G tRNA modification, tRNA expression and mRNA translation efficiency.

**Results:**

The levels of METTL1 and WDR4 are elevated in HCC and associated with advanced tumour stages and poor patient survival. Functionally, silencing METTL1 or WDR4 inhibits HCC cell proliferation, migration and invasion, while forced expression of wild‐type METTL1 but not its catalytic dead mutant promotes HCC progression. Knockdown of METTL1 reduces m^7^G tRNA modification and decreases m^7^G‐modified tRNA expression in HCC cells. Mechanistically, METTL1‐mediated tRNA m^7^G modification promotes the translation of target mRNAs with higher frequencies of m^7^G‐related codons. Furthermore, in vivo studies with *Mettl1* knockin and conditional knockout mice reveal the essential physiological function of Mettl1 in hepatocarcinogenesis using hydrodynamics transfection HCC model.

**Conclusions:**

Our work reveals new insights into the role of the misregulated tRNA modifications in liver cancer and provides molecular basis for HCC diagnosis and treatment.

## Graphical abstract

- m^7^G tRNA modification and its catalytic enzyme METTL1 are elevated in hepatocellular carcinoma (HCC) and associated with poor HCC prognosis.
- The m^7^G tRNAs are essential for tRNA expression and mRNA translation.
- Overactive m^7^G tRNA expression promotes hepatocarcinogenesis in vitro and in hydrodynamic transfection HCC mouse models.

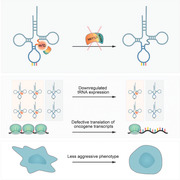

## 1 INTRODUCTION

Primary liver cancer ranks the sixth most common cancers and the fourth leading pathogeny of cancer‐related mortality all over the world.^1^, ^2^ It was estimated that 841 080 new incident cases of liver cancer occurred and 781 631 died in 2018.^1^ Hepatocellular carcinoma (HCC) accounts for 75–80% of primary liver cancers.^1^ Surgical resection and ablation are widely used for the treatment options of early‐stage HCC;^2^ however, more than 70% patients recurred within 5 years.^3^, ^4^ For advanced HCC, transarterial chemoembolization and multi‐target tyrosine kinase inhibitors, including sorafenib and lenvatinib, are recommended, but the efficacy remains limited.^2^ Therefore, better understanding of the molecular mechanisms regulating hepatocarcinogenesis is urgently needed for the development of effective HCC treatment strategies.

HCC initiation and progression are regulated by complex mechanisms, including genetic and epigenetic misregulations.^5^ Recent studies revealed that misregulated RNA modifications often result in aberrant gene expression and are closely related to the developmental diseases and cancers.^6^, ^7^, ^8^ Different RNA species contain posttranscriptional modifications, and the diverse modifications on RNAs play essential functions in RNA metabolism, gene expression regulation and human diseases, including cancers.^9^, ^10^ Therefore, investigation of modifications on RNAs in the regulation of HCC progression could potentially identify regulatory factors and therapeutic targets in HCC.

Transfer RNAs (tRNAs) are the adapter molecules for protein translation in recognizing mRNA codons and bringing the corresponding amino acids. tRNAs are heavily modified and modifications on tRNAs are crucial for tRNA stability, codon recognition and efficient protein synthesis.^7^ Dysregulations of tRNA modification are highly relevant with the development of mitochondrial diseases, neurological disorders and cancers.^7^ N^7^‐methylguanosine (m^7^G) is a highly conserved tRNA modification found in prokaryotes, eukaryotes, as well as in some archaea. The m^7^G tRNA modification is catalyzed by the Trm8p/Trm82p heterodimeric complex in yeast and the corresponding orthologs methyltransferase‐like protein‐1 (METTL1) and WD repeat domain 4 (WDR4) protein in human.^11^ The m^7^G tRNA modification is a non‐essential modification in yeast, but defected m^7^G tRNA modification in mammals is associated with impaired stem cell fate determination^12^ and developmental diseases.^13^, ^14^ Moreover, METTL1 is upregulated in cancers^15^, ^16^ and regulates 5‐Fluorouracil sensitivity in human cancer cells.^17^ Recently, it was reported that METTL1‐mediated m^7^G tRNA modification plays a major part in cancer progression.^18^, ^19^, ^20^ However, the precise roles and regulatory mechanisms of METTL1 and m^7^G tRNA modifications in HCC development and progression remain elusive.

Currently, it was shown that m^7^G tRNA modification as well as its catalyzing enzymes METTL1 and WDR4 protein are markedly upregulated in HCC and inversely related with HCC patient survival. In addition, METTL1‐mediated m^7^G tRNA modification enhances mRNA translation, as well as accelerates HCC progression and tumourigenesis in vitro and in vivo, suggesting that targeting METTL1 and misregulated tRNA modifications could be a promising strategy for HCC treatment.

## 2 METHODS

### 2.1 Patient samples

HCC patients’ clinical samples, including tumour and adjacent liver tissues, were obtained from January 2016 to August 2019 at the First Affiliated Hospital of Sun Yat‐sen University. Patient inclusion criteria are as follows: (1) primary HCC with pathological confirmation; and (2) underwent curative hepatectomy. Exclusion criteria are as follows: (1) received other cancer‐related treatments before hepatectomy; (2) and insufficient pathological samples. Detailed clinical parameters of enrolled patients are provided in Table S1. All patients provided informed consent. Frozen tissues were applied to subsequent RNA or protein extraction. Paraffin‐embedded specimens were used for immunohistochemistry (IHC) analysis. All studies involving human beings were reviewed and approved by the Ethics Committee of the First Affiliated Hospital of Sun Yat‐sen University (Approval number: [2018]072).

### 2.2 Cell culture

The human HCC cell lines purchased from American Type Culture Collection (ATCC, including Huh7, SNU‐449, Hep3B, PLC/PRF/5 and SK‐Hep‐1) or Chinese Type Culture Collection (MHCC97H) were used. Dulbecco's modified Eagle's medium (DMEM, Gibco), Roswell Park Memorial Institute (RPMI 1640, Gibco), foetal bovine serum (FBS, Gibco) and Penicillin‐Streptomycin (PS, Gibco) were used for cell culture. The above human HCC cell lines were cultured in DMEM (Huh7, MHCC97H, Hep3B and SK‐Hep‐1) or RPMI‐1640 medium (SNU‐449 and PLC/PRF/5) supplemented with 10% FBS and 1% PS. The immortalized human liver cell line THLE‐2, which was obtained from ATCC, was cultured in bronchial epithelial cell basal medium and additives (Lonza, Salisbury, MD, USA). Cell culture incubator (Thermo Fisher Scientific, Waltham, MA, USA) was set as 37°C and 5% CO_2_.

### 2.3 Knockdown of METTL1 or WDR4 in HCC cells

The pLKO.1 lentiviral vectors expressing short hairpin RNA (shRNA) against METTL1 and WDR4 were purchased from Horizon Discovery. Inc. The shRNA construct together with packaging plasmid (pCMV‐ΔR8.9) and envelope plasmids (pCMV‐VSVG) were co‐transfected into HEK 293T cells with Lipofectamine 3000 (Invitrogen, Waltham, MA, USA). After 48 h of incubation, the lentiviruses were collected to infect Huh7 and MHCC97H cells with Polybrene (8 μg/ml). After transfection of lentiviruses, puromycin (2.5 and 5.0 μg/ml for Huh7 and MHCC97H, respectively) was used to screen METTL1 or WDR4 knockdown cells for 48 h. For puromycin intake assay, small interfering RNA (siRNA) targeting 3′‐untranslated region of METTL1 (Ribobio, China) was used for METTL1 knockdown. siRNA sequences are listed in Table S2.

### 2.4 Ectopic expression of METTL1, EGFR and LysCTT tRNA in HCC cells

The overall‐length open reading frame of EGFR gene (NM_005228.5) was cloned into pEZ‐Lv201 vector (GeneCopoeia, China) to generate EGFR expression plasmids. The FLAG tag containing METTL1 wild‐type and catalytic dead mutant (aa160‐163, LFPD to AFPA) plasmids (pFLAG‐CMV2) were generated previously.^12^ Three repeated tRNA‐LysCTT gene sequences plus upstream (200 nucleotide sequences) and downstream (200 nucleotide sequences) sequences were cloned into pUC19 vector to generate tRNA‐LysCTT expression plasmids. For overexpression study, Lipofectamine 3000 was used to transfect the corresponding plasmids into the cells.

### 2.5 Immunohistochemistry

IHC was performed as previously described.^18^ After deparaffinization, dehydration, antigen retrieval, blocking and incubation with primary antibody, HCC tissue sections were stained using Diaminobenzidine (DAB) detection. For IHC analysis, Image Pro Plus software was used. The protein levels of METTL1, WDR4 and AFP were determined by the mean density of positive staining calculated from five randomly selected fields. The proportions of Ki67‐positive cells in tumour were calculated from five randomly selected fields. Primary antibodies are listed in Table S3.

### 2.6 RNA isolation and quantitative real‐time polymerase chain reaction

Trizol RNA extraction reagent was used to isolate total RNAs (Ambion, Austin, TX, USA). cDNA was prepared through reverse transcription using Takara PrimeScript RT reagent Kit for subsequent quantitative real‐time polymerase chain reaction (qRT‐PCR) assay. Next, to detect the gene expression, qRT‐PCR was performed using TB Green method following the manufacturer's instruction in a Roche LC480 instrument. The data were evaluated by the 2^–ΔΔCt^ method using β‐actin as a loading control. Primer sequences employed in this study are listed in Table S2.

### 2.7 Northwestern blot, western blot, northern blot and coimmunoprecipitation

Northwestern blot and western blot were both carried out as described previously.^12^ Northern blot was modified as previously reported.^21^ Briefly, for northwestern blotting, after urea‐PAGE electrophoresis, total RNA samples were transferred onto a positively charged nylon membrane. The membrane was crosslinked for 3 min under ultraviolet light and immediately blotted with anti‐m^7^G antibody. Afterwards, the m^7^G signals were detected according to the western blot protocol as previously stated.^12^ For northern blotting, the membrane containing RNAs was blotted with digoxigenin‐labelled probes against U6 snRNA or probes against tRNA‐LysCTT after transfer and crosslink. To demonstrate the interaction between METTL1 and WDR4, coimmunoprecipitation was performed. Briefly, 500 μg proteins were incubated with 1 μg anti‐WDR4 or anti‐IgG plus Protein A/G Magnetic Beads (Thermo Fisher Scientific) with head‐over‐tail rotation overnight at 4℃. Next day, after washing the beads, the IP products were used for western blot. The probe sequences are listed in Table S2. Primary antibodies are listed in Table S3.

### 2.8 Liquid chromatography‐coupled mass spectrometry for the quantitative analysis of tRNA modifications

Liquid chromatography‐coupled mass spectrometry (LC‐MS) was conducted as previously described.^22^, ^23^ Briefly, total RNAs were extracted from the tissues as described above and tRNAs were separated from total RNAs using urea‐PAGE electrophoresis. Then, tRNAs were hydrolyzed to single dephosphorylated nucleosides, followed by deproteinization. Nucleosides were analysed by LC‐MS on Agilent 6460 QQQ mass spectrum analyser following a 1260 HPLC device (Agilent, Santa Clara, CA, USA) and then the raw data were derived by Agilent Qualitative Analysis software. Multi reaction monitoring peaks of each modified nucleoside were derived and normalized to quantity of tRNAs. The percentage of tRNA modification was defined as the ratio between the normalized peak area of tRNA modification and the sum of the normalized peak area of all nucleosides detected.

### 2.9 Clone formation, cell proliferation, viability and invasion assays

For clone formation assay, 1000 cells/well were seeded in six‐well plates with fresh medium. After 2 weeks, colony numbers were recorded by staining with .5% crystal violet. For viability assay, 2000 cells/well were seeded in 96‐well plates with fresh medium. Cell viabilities were evaluated at 0, 24, 48, 72 and 96 h using Cell Counting Kit‐8 (Dojindo, Japan) according to the manufacturer's instructions.

Cells were incubated with mitomycin C (Selleck, Selleck, WA, USA) (10 μg/ml final concentration) for 1 h before migration or invasion assay. A total of 5 × 10^4^ cells resuspended in 500 μl serum‐free fresh medium were added to the upper chamber of a transwell insert (Corning Falcon), which then was placed on a well containing fresh medium with 20% foetal bovine serum to induced cell migration. For invasion assay, cells were seeded into a Matrigel‐coated chamber (Becton Dickinson, ‎Franklin Lakes, NJ, USA) with the same condition in migration assay. The cells transferred to the outside surface were counted by staining with .5% crystal violet after 48 or 72 h to analyse the cell migration and invasion.

### 2.10 Cell apoptosis and cell cycle assays

Cell apoptosis was detected by Annexin V‐FITC Apoptosis Detection Kit (KeyGEN BioTECH, China) following the manufacturer's instruction and then analysed with CytoFLEX (Beckman Coulter, Brea, CA, USA). Cell cycle assay was carried out using PI/RNase Staining Kit (Dojindo) and FITC BrdU Flow Kit (BD Pharmigen, San Diego, CA, USA) in accordance with the manufacturer's instruction. Flow cytometry analysis was performed using CytoFLEX (Beckman Coulter).

### 2.11 Polysome profiling

Polysome profiling was carried out following the protocol as previously stated.^12^ A 10‐50% sucrose density gradient was prepared in a SW41 ultracentrifuge tube (Beckman Coulter) using a peristaltic pump. Polysome lysis buffer was prepared by the reagents as follows: 50 mM MOPS, 150 mM NaCl, 15 mM MgCl_2_, .5% Triton X‐100, 100 μg/ml cycloheximide, 1 mg/ml Heparin, 2 mM PMSF, 200U/ml RNase inhibitor and 1 mM benzamidine. After incubation, cells were rinsed by cold PBS plus 100 μg/ml cycloheximide three times immediately, then lysed using polysome lysis buffer for 10 min on the ice. The supernatant was laid on the top of sucrose gradients carefully after centrifuging cell lysis (13 000 *g*, 10 min, 4°C), and subsequently centrifuged in ultracentrifuge Optima XE‐100 (Beckman Coulter) at 36 000 rpm at 4°C for 3 h. Samples separated after ultracentrifugation were fractionated immediately at a speed of .75 ml/min via Brandel BR‐188 Density Gradient Fractionation Device. OD254 values were continually monitored and recorded during fractionation.

### 2.12 Puromycin intake assay

To monitor the new protein translation rate in HCC, puromycin intake assay was carried out based on previous protocol.^24^ Briefly, cells were incubated with 1 μM puromycin for 30 min in cell culture incubator at 37°C. After incubation, cells were lysed to extract protein and western blot was performed to detect the level of puromycin incorporation using anti‐puromycin antibody.

### 2.13 tRNA m^7^G reduction and cleavage sequencing

For analyzing tRNA m^7^G methylation and tRNA expression level, tRNA m^7^G reduction and cleavage sequencing (TRAC‐seq) was conducted following previous protocol with some modifications.^12^, ^25^, ^26^, ^27^ Briefly, small RNAs were isolated from total RNAs using mirVana™ miRNA Isolation Kit (Invitrogen, Waltham, MA, USA), followed by recombinant wild‐type and mutant (D135S) ALKB proteins treatment.^27^ The ALKB proteins were home made with Addgene plasmids (pET30a‐AlkB, Cat# 79050 and pET30a‐AlkB‐D135S, Cat# 79051). Next, the ALKB‐treated RNAs were reduced by .1 M NaBH_4_ for 30 min at 4°C away from light. Then, to induce m^7^G‐modified‐site cleavage, NaBH_4_‐reduced RNAs were cut off by acetate‐aniline mixture (H_2_O: acetic acid: aniline, 7:3:1) for 2 h at ambient temperature away from light. After cleavage, the RNAs were purified with Oligo Clean & Concentrator™ kit (Zymo Research, Irvine, CA, USA). Finally, the purified RNAs were applied to constructing cDNA library by NEBNext Multiplex Small RNA Library Prep Set for Illumina (New England BioLabs, Ipswich, MA, USA) and sequenced via Illumina Nextseq 500.

### 2.14 TRAC‐seq data analysis

TRAC‐seq data analysis was performed following previous instructions with some modifications.^12^, ^26^ After adaptor trimming and quality filtering, Bowtie2 was applied to aligning the sequencing reads to the reference genome (hg38 for human or mm10 for mouse downloaded from GtRNAdb database) plus the set of mature tRNA sequences predicted from tRNAscan‐SE. The tRNA sequences and Bowtie indexes of human and mouse could be downloaded at the website of https://github.com/rnabioinfor/TRAC‐Seq/tree/master/bowtie_index. Mature tRNA sequences were deleted the predicted introns and then added ‘‘CCA’’ to the 3′ end. During the mapping process, the best alignment of reads with no more than 50 hits are reported. For tRNA m^7^G analysis, after mapping clean reads to mature tRNA sequences, Bedtools was used to process the alignments and record the read depth of cleavage site i together with the number of reads staring at site i. The cleavage ratio of site i was defined as the ratio between the number of reads (site i) and the read depth (site i). Then, the cleavage score of sites was calculated as: Cleavage score_i _= log 2(Cleavage ratio_treat_)/log 2(Cleavage ratio_non‐treat_). The positions 46–48 along with a cleavage score more than 4 and the cleavage ratio more than .1 were defined as the m^7^G‐modified sites.

In the analysis of tRNA expression, the read count of specific tRNA was evaluated from ALKB‐treated tRNA sequencing data as previously described.^25^ Briefly, all best matches were generated by the scoring function of Bowtie2. Reads with the same mapping scores to tRNA gene loci and predicted mature tRNA sequences were mapped exclusively to mature tRNAs. Then, the read counts of each tRNA were normalized using RPKM (reads per kilobase per million) method. tRNAs with RPKM > 10 000 in expression level were included for analysis.

### 2.15 m^7^G methylated tRNA immunoprecipitation qPCR

Anti‐m^7^G methylated tRNA immunoprecipitation qPCR was carried out as we previously reported.^12^ Briefly, 20 μg total RNAs were incubated with 2 μg anti‐m^7^G antibody with rotation for 2 h at 4°C. Next, 100 μl Protein A/G Magnetic Beads were washed and applied to the reaction mixture, which subsequently incubated with rotation for 2 h at 4°C to purify the m^7^G‐modified RNAs. After incubation, the beads were rinsed extensively and mixed with Trizol reagent to extract the RNAs. The precipitated and input RNAs were demethylated by homemade recombinant wild‐type and mutant (D135S) ALKB proteins to abolish the dominant methylations.^27^ After demethylation treatment, the ALKB‐treated RNAs were purified for subsequent qPCR using Oligo Clean & Concentrator™ kit.

### 2.16 Polyribosome‐associated mRNA sequencing and qPCR

To evaluate the translation efficiency (TE) of specific genes, polyribosome‐associated mRNA sequencing and qPCR were conducted following a published protocol with some modifications.^28^ Briefly, cells were rinsed using pre‐cooling PBS containing 100 μg/ml cycloheximide and subsequently lysed with cell lysis buffer [1% Triton X‐100 diluted with ribosome buffer (RB buffer), containing 20 mM HEPES‐KOH (pH 7.4), 200 mM KCl, 15 mM MgCl_2_, 2 mM dithiothreitol along with 100 μg/ml cycloheximide]. After 30 min incubation on ice, cell lysates were centrifuged (16 200 *g*, 10 min, 4°C). The supernatants were divided into two parts: 10% of the supernatants were saved as the input control for the subsequent qPCR or sequencing and the remaining supernatants were added to the surface of 35 ml 30% sucrose solution with RB buffer. Polyribosomes were collected through ultra‐centrifugation (174 900 *g*, 5 h, 4°C) in a SW32 rotor (Beckman Coulter). Next, total RNAs and polyribosome‐RNAs were extracted for subsequent qPCR and sequencing.

Polyribosome‐associated mRNA‐seq were conducted by the Beijing Genomics Institute (BGI‐shenzhen, China) on BGISEQ‐500 platform as reported previously.^29^ Short reads alignment procedure Bowtie2^30^ was utilized for mapping reads to the human reference genome (GRCh38). The gene expression level was standardized as FPKM (fragments per kilobase of transcript per million mapped reads). For polyribosome‐associated mRNA‐seq data analysis, TE was determined as: TE = (FPKM in polyribosome‐associated mRNA‐seq) / (FPKM in RNA‐seq).

### 2.17 Pathway enrichment analysis

Pathway enrichment analysis through Wikipathway was performed at the website of http://www.webgestalt.org/ using an online analysis tool WebGestalt. Over‐representation analysis was selected. Pathways with false discovery rate less than .05 were considered as significantly enriched.

### 2.18 TCGA data analysis

Raw data (including read counts, copy number and DNA methylation) and clinical information of the Cancer Genome Atlas Liver Cancer Hepatocellular carcinoma (TCGA‐LIHC) dataset were derived from the TCGA data portal (https://portal.gdc.cancer.gov/). Raw counts data were normalized using transcripts perkilobase million (TPM) for subsequent analysis. Comparison analysis was conducted using log_2_(TPM). Survival analysis was conducted between METTL1‐ or WDR4‐high and METTL1‐ or WDR4‐low groups. DNA copy number data were analysed using GISTIC 2.0 software. The copy number more than 1 was defined as amplification, while less than −1 was defined as loss.

### 2.19 Luciferase reporter assay

For function analysis of tRNA‐LysCTT, dual reporter plasmid, including firefly luciferase (F‐luc) coding sequence and Renilla luciferase (R‐luc) coding sequence, was established on the basis of pmirGlo luciferase expression vector (Promega, Madison, WI, USA). Five repeats of AAG codon sequence (5X AAG) were introduced into the upstream of F‐luc coding sequence. Five hundred nanograms of 5X AAG (Lys) or control reporter plasmids were transfected into Huh7 or MHCC97H cells using Lipofectamine 3000 in a six‐well plate. After 48 h, the cells were assayed by Dual‐Glo Luciferase Assay system (Promega) in a 96‐well plate. R‐luc was used for the normalization of F‐luc activity.

### 2.20 Subcutaneous implantation mouse model

Nude mice (4‐week‐old, male) were acquired from Model Animal Research Center of Nanjing University. To establish the xenograft model, mice were classified into control and METTL1 or WDR4 knockdown groups randomly and injected with corresponding cancer cells (.1 ml PBS for each mouse). Tumours were examined with a caliper every 4 days and the long diameter (a) and the short diameter (b) of tumours were recorded. Then, the tumour size (*V*) was calculated by the equation of *V* = ab^2^/2. When the tumour size and health status met the institutional euthanasia criteria, the mice would be euthanized and tumour tissues would be harvested. All animal procedures were authorized by the Institutional Animal Care and Use Committee of Sun Yat‐sen University (Approval number: SYSU‐IACUC‐2020‐000203).

### 2.21 Mettl1 knockin mouse model

The Mettl1 knockin mice were generated using the CRISPR‐Cas9 technology in the Beijing Biocytogen Co., Ltd. To generate transgenic mice stably overexpressing Mettl1, a CAG promoter‐driven loxP‐Stop‐loxP‐Mettl1‐IRES‐tdTomato fragment was inserted into the Rosa26 locus. After screening F0 mice, F1 heterozygous mice were crossed with CMV‐Cre mice to generate stable Mettl1 knockin mice (CMV‐Cre+; Mettl1^KI^) for subsequent experiments. The CMV‐Cre positive wild‐type mice (CMV‐Cre+; Mettl1^wt^) were used as controls.

### 2.22 Alb‐CreERT2‐mediated Mettl1 conditional knockout mouse model

The Mettl1^flox^ mice were generated using the CRISPR‐Cas9 technology in the Beijing Biocytogen Co., Ltd. Alb‐CreERT2 mice were crossed with Mettl1^flox/wt^ mice to obtain inducible liver‐specific Mettl1 knockout mice (Alb‐CreERT2+; Mettl1^flox/flox^). The Alb‐CreERT2 positive wild‐type mice (Alb‐CreERT2+; Mettl1^wt/wt^) were used as controls. To induce Mettl1 knockout, tamoxifen (1 mg per mouse) was injected intraperitoneally once a day for 5 consecutive days.

### 2.23 Hydrodynamics transfection

To induce hepatocarcinogenesis mouse model, we performed hydrodynamic transfection through sleeping beauty (SB) transposon system as previously described.^33^ Briefly, 25 μg myr‐AKT1 (Addgene, Plasmid#31789) and 25 μg N‐RasV12 (Addgene, Plasmid#20205) along with 2 μg SB transposase were diluted in 2.5 ml saline, and then injected into WT or Mettl1‐KI C57BL/6 mice (8–12 weeks old) in 5–7 s via the lateral tail vein. At 4–6 weeks after hydrodynamic transfection, livers were harvested and used for the analysis of HCC tumourigenesis.

### 2.24 Statistical analysis

Statistical analysis methods were illustrated in separate method sections or figure legends. Graphpad prism 8.0 and R version 3.5.0 were used to conduct all statistical analyses.

## 3 RESULTS

### 3.1 m^7^G tRNA modification and its catalyzing enzyme METTL1/WDR4 complex are elevated in HCC

**FIGURE 1:**
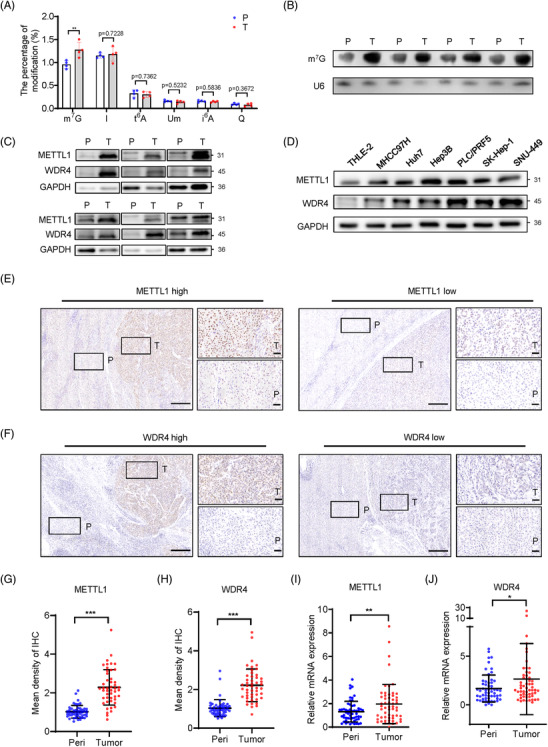
m^7^G tRNA modification and its catalyzing enzyme components METTL1 and WDR4 are elevated in HCC. (A) The percentage of m^7^G tRNA modification and other tRNA modifications in four pairs of HCC tissues and corresponding normal liver tissues identified by liquid chromatography‐coupled mass spectrometry. Paired Student's *t* test was used (*n* = 4). (B) Northwestern blot of m^7^G tRNA modification in four pairs of HCC tissues and corresponding normal liver tissues. U6 northern blot serves as a loading control. (C) Western blot of METTL1 and WDR4 in six pairs of HCC tissues and corresponding peritumoural tissues. GAPDH serves as a loading control. (D) Western blot of METTL1 and WDR4 in HCC cell lines. A normal liver cell line THLE‐2 was used as a normal control and GAPDH serves as a loading control. (E, F) Representative images of METTL1 (E) and WDR4 (F) IHC staining in HCC specimens. The mean density of IHC staining less than median was defined as low, while more than median was defined as high. Scale bar, 250 μm. (G, H) Quantification of METTL1 (G) and WDR4 (H) IHC staining intensity in HCC specimens. Paired Student's *t* test was used (*n* = 48). (I, J) qRT‐PCR analysis of METTL1 (I) and WDR4 (J) mRNA expression in HCC specimens. Wilcoxon signed rank test was used (*n* = 57). Data presented as mean ± SD. *p < .05, **p < .01, ***p < .001 by Student's *t* test or the Mann–Whitney U test unless specified. Abbreviations: IHC, immunohistochemistry; I, inosine; i^6^A, N^6^‐isopentenyladenosine; P, peri‐tumour tissue; Peri, peri‐tumour tissue; Q, queuosine; T, tumour tissue; t^6^A, N^6^‐threonylcarbamoyladenosine; Um, 2′‐O‐methyluridine

To identify the tRNA modifications that regulate HCC progression, we performed tRNA modification profiling using LC‐MS and revealed that m^7^G tRNA modification is significantly upregulated in HCC samples compared with normal liver samples (Figure 1A). In addition, our northwestern blot analysis also validated the elevated m^7^G tRNA modification in HCC tissues (Figure 1B). Consistent with these, our western blot showed that m^7^G tRNA methyltransferase complex proteins METTL1 and WDR4^12^, ^31^ were upregulated in HCC tumour tissues and cell lines (Figure 1C, D). Using IHC staining and qRT‐PCR assays, we further confirmed that the protein and mRNA expression levels of both METTL1 and WDR4 were significantly elevated in HCCs compared to the corresponding peritumoural tissues (Figure 1E–J). To study the relationship between METTL1/WDR4 expression and HCC prognosis, we analysed a large‐cohort HCC sample using the TCGA LIHC dataset. Consistent with our results, METTL1 and WDR4 mRNA expression levels are significantly elevated in HCCs (Figure S1A, B). Moreover, high METTL1 or WDR4 expression correlates with advanced tumour stages, vascular invasion statuses and poor HCC patient survival (Figure S1C‐F). Analysis of the TCGA‐LIHC data revealed that 15.4% of HCC patients present the amplification of METTL1 DNA (Figure S1G) and the METTL1 mRNA expression is significantly elevated in patients with METTL1 DNA amplification (Figure S1H). In addition, we also found that METTL1 DNA methylation level is negatively correlative to the METTL1 mRNA expression, suggesting that DNA methylation may also play a major part in the overactivation of METTL1 (Figure SI‐SJ). These data suggested that copy number gain and misregulated DNA methylation lead to the upregulation of METTL1 in liver cancer.

### 3.2 METTL1 knockdown reduces m^7^G tRNA modification and suppresses HCC progression

**FIGURE 2:**
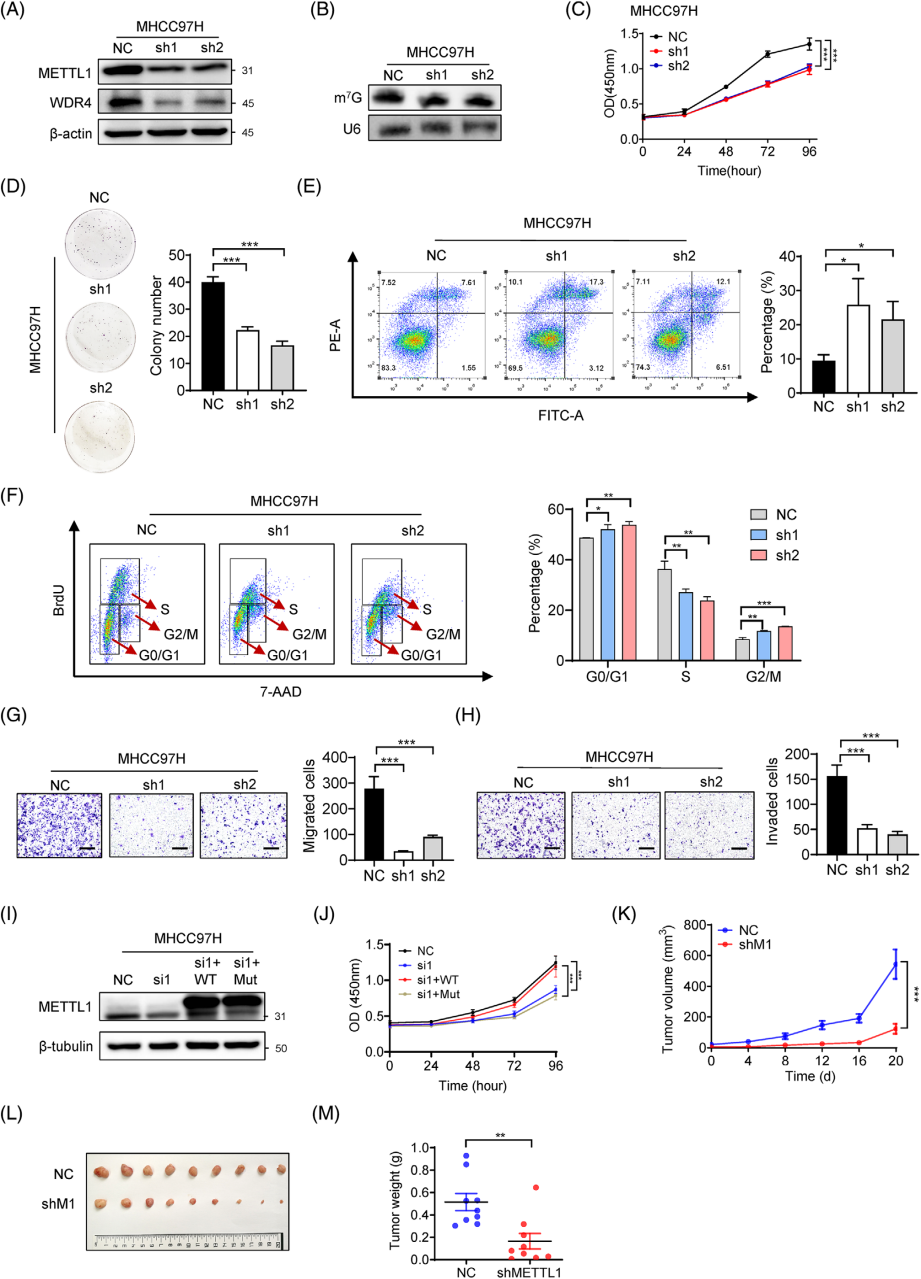
Inhibition of METTL1 impairs HCC progression in vitro and in xenograft model. (A) The knockdown effect of METTL1 in MHCC97H cells was confirmed by western blot. (B) The downregulation of m^7^G tRNA modification was confirmed by northwestern blot. (C) CCK‐8 assay of METTL1 knockdown and control MHCC97H cells. Data presented as mean ± SD (six technical replicates). (D) Representative images and quantification of clone formation in METTL1 depleted and control MHCC97H cells. Data presented as mean ± SD (three technical replicates). (E) Representative images and quantification of cell apoptosis assays in MHCC97H cells with or without METTL1 knockdown. Data presented as mean ± SD (three technical replicates). (F) Cell cycle analysis and quantification of METTL1 depleted and control MHCC97H cells. Data presented as mean ± SD (three technical replicates). (G) Representative images and quantification of migration in METTL1 depleted and control MHCC97H cells. Scale bar, 500 μm. Data presented as mean ± SD (three technical replicates). (H) Representative images and quantification of invasion in METTL1 depleted and control MHCC97H cells. Scale bar, 500 μm. Data presented as mean ± SD (three technical replicates). (I) Validation of the rescue of METTL1 by western blot in MHCC97H cells. siMETTL1‐1 was used. (J) CCK‐8 assay of METTL1 knockdown MHCC97H cells with rescue expression of wild‐type METTL1 or the mutant. (K) Growth of subcutaneous transplanted tumours in NC and shMETTL1 group. Tumour sizes were measured every 4 days. Data presented as mean ± SEM (*n* = 9). shMETTL1‐2 was used. (L) Overview of subcutaneous transplanted tumours in NC and shMETTL1 group. (M) Tumour weights formed in NC and shMETTL1 group at the time of sacrifice. Data presented as mean ± SEM (*n* = 9). *p < .05, **p < .01, ***p < .001 by Student's *t* test, one‐way ANOVA or the Mann–Whitney U test unless specified. All the in vitro assays were biologically repeated for three times. Abbreviations: Mut, mutant METTL1; NC, negative control; sh1, shMETTL1‐1; sh2, shMETTL1‐2; shM1, shMETTL1; si1, siMETTL1‐1; WT, wild‐type METTL1

Considering that METTL1 and WDR4 act together as a stable m^7^G tRNA methyltransferase complex (Figure S2), we next studied the function of m^7^G tRNA modification in HCC progression. Our data revealed that knockdown of METTL1 in HCC cell lines Huh7 and MHCC97H resulted in decreased m^7^G tRNA modifications (Figure 2A, B and Figure S3A‐C), and inhibited the growth (Figure 2C and Figure S3D), colony formation (Figure 2D and Figure S3E), cell cycle progression (Figure 2F and Figure S3F), migration and invasion capacities (Figure 2G, H and Figure S3H, I) in both Huh7 and MHCC97H cells. Moreover, METTL1 knockdown significantly increases apoptosis in both Huh7 and MHCC97H cells (Figure 2E and Figure S3G). Interestingly, overexpression of wild‐type METTL1 but not its catalytic inactive mutant could rescue cell proliferation in siMETTL1 HCC cells (Figure 2I, J and Figure S3J, K), which suggests that the catalytic activity is essential for METTL1 to promote HCC progression. Furthermore, subcutaneous xenograft model was constructed to study the effect of METTL1 knockdown on cancer cell growth in vivo. Consistently, the shMETTL1 group showed slower tumour growth, reduced tumour size and weight compared to the NC group (Figure 2K–M ). Overall, our results revealed that METTL1 plays an important function in regulating tRNA modification and hepatocarcinogenesis in vitro and in subcutaneous implantation model.

### 3.3 m^7^G tRNA modification regulates tRNA expression and affects global mRNA translation in HCC

**FIGURE 3:**
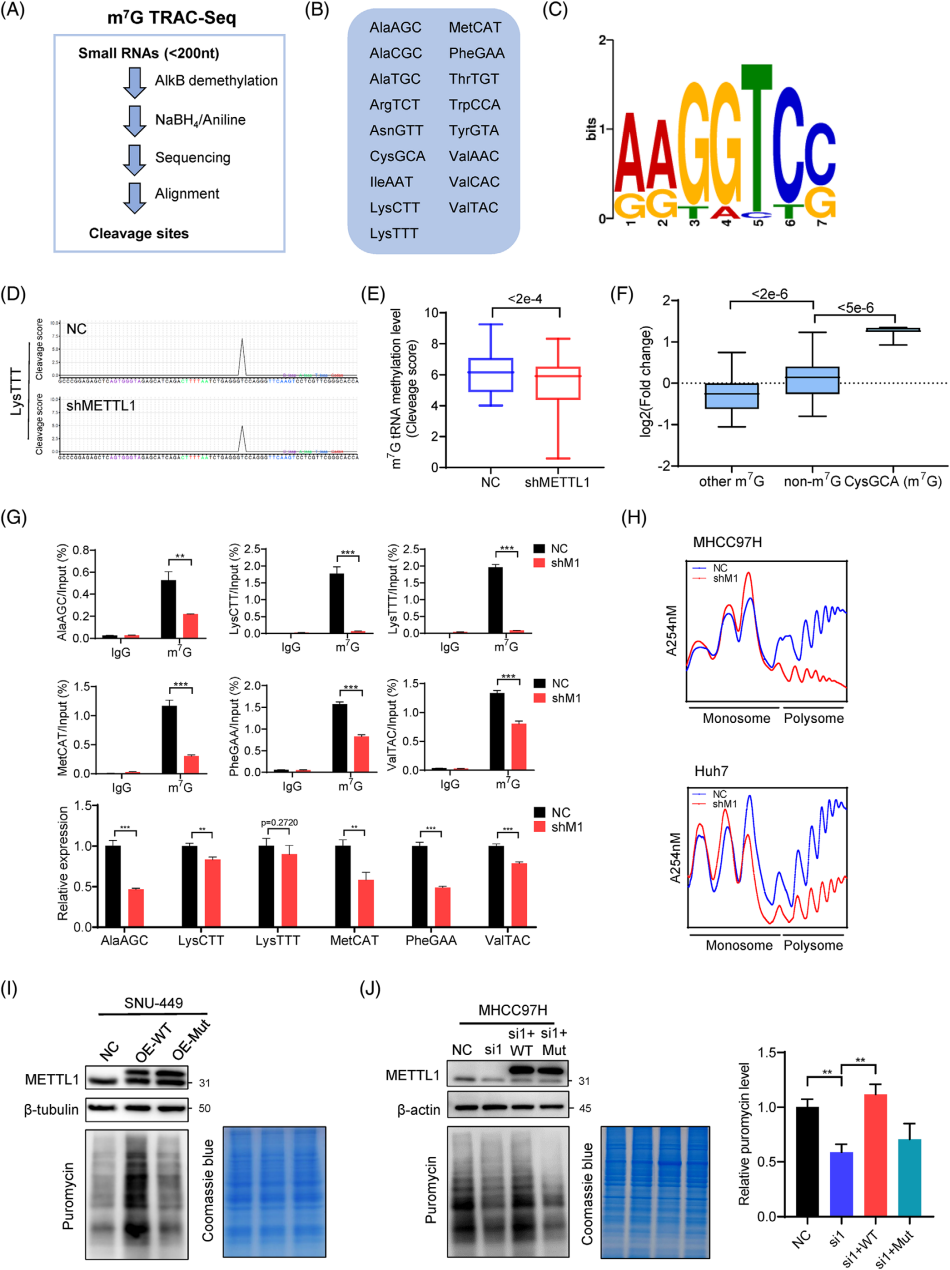
METTL1 regulates m^7^G tRNA methylome, tRNA expression and global mRNA translation. (A) Flowchart of m^7^G TRAC‐Seq. (B) List of m^7^G‐modified tRNA identified in MHCC97H cells. (C) Sequence motif in the m^7^G sites identified by TRAC‐seq. (D) Representative images of cleavage scores of indicated tRNA in MHCC97H cells with or without METTL1 knockdown. (E) Global m^7^G tRNA methylation level of MHCC97H cells with or without METTL1 knockdown. Wilcoxon signed‐rank test was used. (F) Expression level of m^7^G‐modified and non‐m^7^G‐modified tRNAs revealed by TRAC‐seq. Fold change was calculated as the ratio of tRNA expression level of shMETTL1 group to the control group. (G) Validation of the downregulated expression of m^7^G‐modified tRNAs upon METTL1 depletion by m^7^G methylated tRNA immunoprecipitation qPCR. Relative expression of specific tRNA was obtained using input samples and U6 served as an internal control. The shMETTL1‐2 was used in this experiment (three technical replicates). (H) Polysome profiling of Huh7 and MHCC97H with or without METTL1 knockdown. shMETTL1‐2 was used in this experiment. (I) Global translation of SNU‐449 cells with overexpression of wild‐type or mutant METTL1. Coomassie brilliant blue staining of the gel was used as control. (J) Puromycin intake assay of siMETTL1 MHCC97H cells rescued by wild‐type METTL1 or its catalytic dead mutant. Coomassie brilliant blue staining of the gel was used as control. Quantitation of the bands was showed (three biological replicates). siMETTL1‐1 was used. Data presented as mean ± SD. *p < .05, **p < .01, ***p < .001 by Student's *t* test or the Mann–Whitney U test unless specified. Abbreviations: Mut, mutant METTL1; NC, negative control; shM1, shMETTL1; si1, siMETTL1‐1; WT, wild‐type METTL1

To understand the mechanisms underlying m^7^G tRNA modification in the regulation of HCC progression, we used m^7^G site‐specific tRNA reduction and cleavage‐sequencing (TRAC‐seq)^12^, ^26^ method to identify global m^7^G tRNA modifications in MHCC97H cells (Figure 3A). Our TRAC‐seq data identified a total of 17 tRNAs containing m^7^G modifications in MHCC97H cells (Figure 3B, C and Figure S4). METTL1 knockdown significantly reduced m^7^G signal in the m^7^G‐modified tRNAs (Figure 3D, E). We next analysed the expression of tRNAs and found that, except for the tRNA CysGCA, the majority of m^7^G‐modified tRNAs are downregulated upon METTL1 depletion (Figure 3F). Our anti‐m^7^G MeRIP‐qPCR data confirmed that modification and expression levels of m^7^G‐modified tRNAs are downregulated upon METTL1 depletion (Figure 3G), further supporting the function of METTL1 in catalyzing m^7^G tRNA modification and tRNA expression. We next overexpressed METTL1 in SNU‐449, an HCC cell line with low METTL1 expression, to further study its role in m^7^G tRNA modification (Figure S5A). Our TRAC‐seq data revealed that overexpression of METTL1 increases m^7^G tRNA methylation level (Figure S5B, C) and upregulates the majority of m^7^G tRNAs expression (Figure S5D, E). In summary, our results revealed that METTL1 regulates m^7^G tRNA modification and expression in HCC cells.

Since tRNAs are adaptors for mRNA translation, we further studied the effects of m^7^G tRNA modification on translation in HCC. We performed polysome profiling and found that METTL1 depletion caused a reduction of polyribosome peak in both Huh7 and MHCC97H cell lines (Figure 3H), suggesting the decreased global mRNA translation in METTL1‐knockdown cells. To validate the results of polysome profiling, we performed puromycin intake assay, another assay to measure mRNA translation activity. Our data showed that overexpression of METTL1 in SNU‐449 cells increased m^7^G tRNA modification (Figure S5F, G) and promoted the new protein synthesis in the puromycin intake assays, while forced expression of catalytic dead METTL1 mutant had little function in tRNA modification and mRNA translation (Figure 3I). On the other hand, METTL1 depletion caused a decrease of puromycin‐labelled newly synthesized protein in MHCC97H cells (Figure 3J), and overexpression of wild‐type METTL1 but not its catalytic inactive mutant could rescue protein synthesis in siMETTL1 MHCC97H cells (Figure 3J), suggesting that METTL1 promotes mRNA translation through m^7^G tRNA modification. We further knocked down METTL1's interacting partner WDR4 and found that WDR4 depletion also resulted in decreased m^7^G tRNA modification and impaired mRNA translation (Figure S6A‐C). Overall, these results demonstrated the important function of METTL1 and m^7^G tRNA modification in the regulation of mRNA translation in HCC.

### 3.4 METTL1 regulates mRNA translation in m^7^G tRNA decoded codon‐dependent manner

**FIGURE 4:**
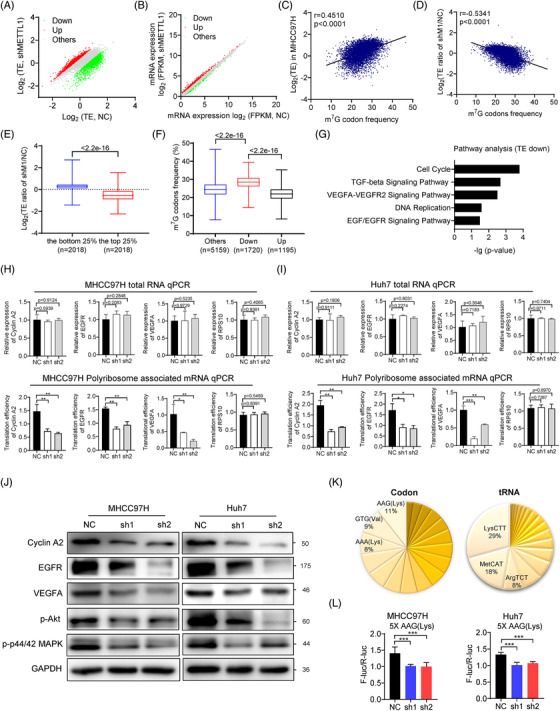
m^7^G tRNA modification regulates HCC mRNA translation in a codon‐dependent manner. (A) Scatterplot of translation efficiency (TE) in the MHCC97 cells with or without METTL1 knockdown. TE was calculated as the ratio of the polyribosome signals to the input signals. (B) Scatterplot of mRNA expression in the MHCC97 cells with or without METTL1 knockdown. (C) Correlation of m^7^G‐related codons frequency and translation efficiency in MHCC97H. Pearson correlation analysis was used. (D) Correlation of m^7^G‐related codons frequency and translation ratio. Translation ratio was calculated as the ratio of the translation efficiency of shMETTL1 group to the control group. Pearson correlation analysis was used. (E) Translation ratio of mRNAs in low (*n* = 2018) and high (*n* = 2018) m^7^G‐related codon frequency groups. (F) Frequencies of m^7^G‐related codons in TE‐decreased genes (*n* = 1720), TE‐increased genes (*n* = 1195) and other genes (*n* = 5159). (G) Pathway analysis using the TE‐decreased genes upon METTL1 knockdown. (H, I) Relative expression and translation efficiency (TE) of Cyclin A2, EGFR and VEGFA mRNA in METTL1 depleted and control MHCC97H (H) and Huh7 (I) cells. β‐Actin was used as an internal control. TE was calculated as the ratio of the polyribosome signals to input signals. RPS10 was used as a negative control. Data presented as mean ± SD (three technical replicates). (J) Western blot of Cyclin A2, EGFR, VEGFA, p‐Akt and p‐p44/42 MAPK in METTL1 depleted and control Huh7 and MHCC97H cells. GAPDH was used as a loading control. (K) Left, m^7^G tRNAs decoded‐codons frequency of TE down mRNAs identified by polyribosome‐mRNA‐seq. Right, the expression profile of m^7^G tRNAs in MHCC97H cells. (L) 5X AAG (Lys) codon sequences were inserted in the front of firefly luciferase coding region. Depletion of METTL1 resulted in decreased luciferase activity compared to that in the controls in Huh7 and MHCC97H cells. The control reporter without any insertion was used to normalize the translation differences. Data presented as mean ± SD (three technical replicates). *p < .05, **p < .01, ***p < .001 by Student's *t* test, one‐way ANOVA or the Mann–Whitney U test unless specified. Polyribosome‐mRNA‐seq was biologically repeated for three times. All the in vitro assays were biologically repeated for three times. Abbreviations: NC, negative control; sh1, shMETTL1‐1; sh2, shMETTL1‐2; TE, translation efficiency

To study the function of m^7^G tRNA modification, we performed polyribosome‐associated mRNA sequencing (polyribosome‐mRNA‐seq) and RNA sequencing (RNA‐seq) using the METTL1 depleted and control MHCC97H cells. Our data revealed that METTL1 knockdown resulted in dramatic changes of TEs but had relatively modest effect on the mRNA expression levels (Figure 4A, B). To determine the correlation between TE and the frequency of m^7^G‐modified tRNAs decoded codons (m^7^G codons) in mRNAs, we plotted all the mRNAs based on their m^7^G codon frequencies and TEs, our data showed that the TEs of mRNAs are positively correlated to the frequencies of m^7^G codons in MHCC97H cells (Figure 4C). Moreover, the TEs of mRNAs with higher m^7^G codons are preferentially downregulated in the METTL1‐depleted cells (Figure 4D, E). On the other hand, codon frequency analysis uncovered that mRNAs with decreased TEs in the METTL1 knockdown cells have significantly higher percentage of m^7^G codons (Figure 4F), suggesting that m^7^G tRNA modification and the frequency of m^7^G codons cooperatively regulate mRNA translation in HCC cells. In summary, our results revealed that METTL1 knockdown decreases m^7^G tRNA modification and expression, which leads to impaired mRNA translation in a codon frequency‐dependent mechanism.

Pathway analysis revealed that genes with decreased TE are significantly associated with cell cycle processes and EGFR signalling pathway (Figure 4G). Our polyribosome‐associated mRNA‐qPCR assay confirmed the decreased TE of Cyclin A2, EGFR and VEGFA mRNAs in METTL1‐knockdown cells (Figure 4H, I). In consistent with this, the decreased TEs resulted in the reduced Cyclin A2, EGFR and VEGFA protein expression levels (Figure 4J). Further, our data revealed the reduced signalling activities of p‐Akt and p‐p44/42 MAPK, which are both the downstream of EGF/EGFR and VEGFA/VEGFR1 signalling pathways, suggesting that METTL1 knockdown decreased the mRNA translation of EGF/EGFR and VEGFA/VEGFR1 signalling pathway components, reduced the downstream signalling activities, and therefore inhibited proliferation and metastasis in HCC (Figure 4J). To support that defected translation of cell cycle genes in METTL1‐depleted cells causes cell cycle arrest, we induced METTL1 knockdown using siRNA and found that TE of Cyclin A2 decreased at 36 h after siRNA transfection, while the G2/M cell cycle arrest appeared at 48 h after siRNA transfection (Figure S7), suggesting that METTL1 knockdown inhibits the translation of Cyclin A2, which then leads to cell cycle arrest.

To confirm that m^7^G tRNA modification mediates METTL1's function in mRNA translation regulation, we established a luciferase reporter that contains five repeats of AAG codons in the front of luciferase coding sequence.^32^ Codon AAG (Lys) was used because its high frequency among all codons of the TE down mRNAs and its corresponding tRNA LysCTT is highly abundant (Figure 4K). Our data showed that METTL1 knockdown markedly inhibits the tRNA‐LysCTT‐mediated translation of luciferase reporter mRNA (Figure 4L).

**FIGURE 5:**
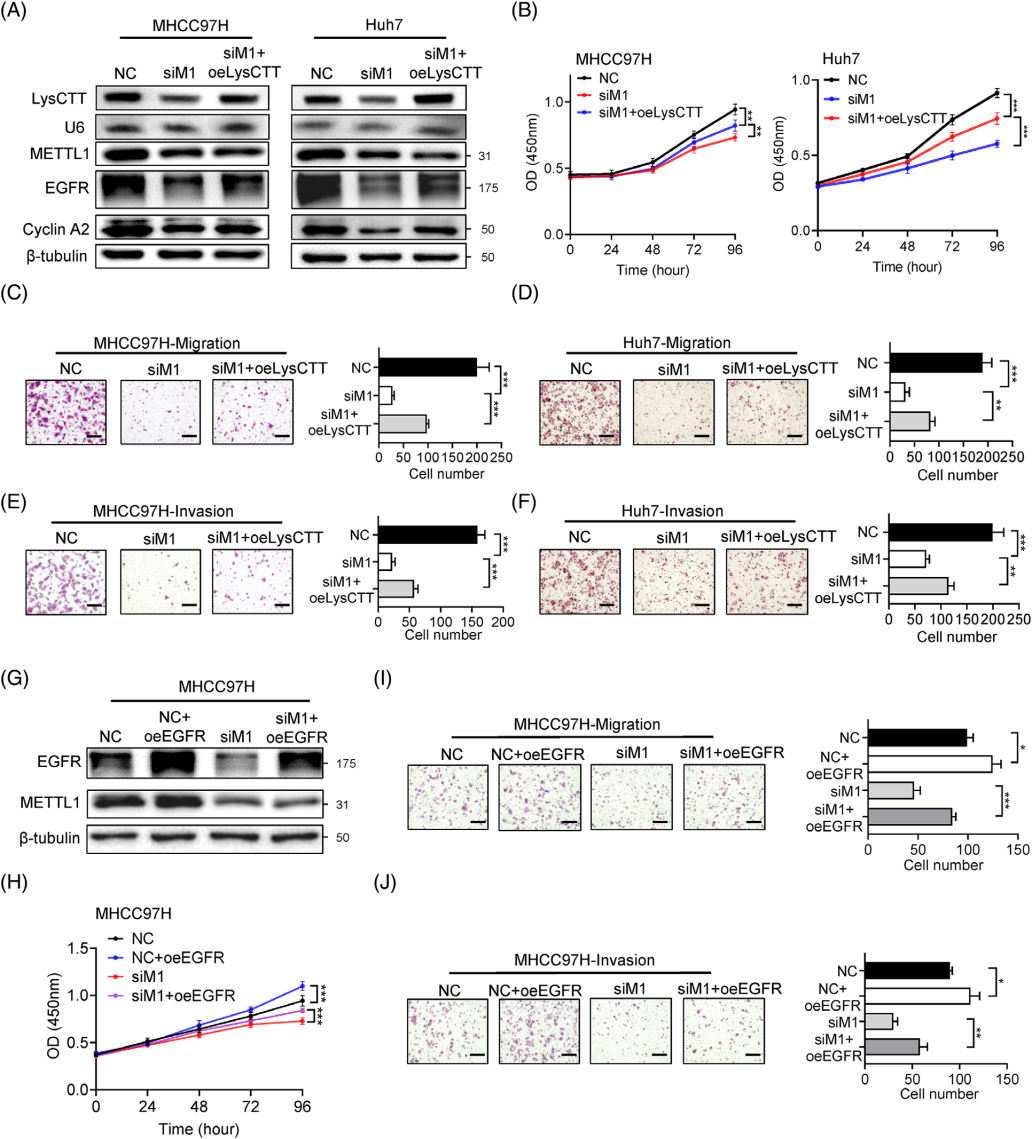
Overexpression of LysCTT and EGFR rescues HCC malignant phenotype. (A) Validation of the LysCTT, METTL1, EGFR, Cyclin A2 expression and METTL1 depletion by northern and western blots in MHCC97H and Huh7 cells. (B) CCK‐8 assay of METTL1 knockdown MHCC97H and Huh7 cells with or without overexpression of LysCTT. Data presented as mean ± SD (six technical replicates). (C, D) Representative images and quantification of migration in METTL1‐knockdown MHCC97H (C) and Huh7 (D) cells with or without overexpression of LysCTT. Scale bar, 500 μm. Data presented as mean ± SD (three technical replicates). (E, F) Representative images and quantification of invasion in METTL1‐knockdown MHCC97H (E) and Huh7 (F) cells with or without overexpression of LysCTT. Scale bar, 500 μm. Data presented as mean ± SD (three technical replicates). (G) Validation of the EGFR overexpression and METTL1 depletion by western blot in MHCC97H cells. (H) CCK‐8 assay of METTL1 knockdown MHCC97H cells with or without overexpression of EGFR. Data presented as mean ± SD (six technical replicates). (I) Representative images and quantification of migration in METTL1‐knockdown MHCC97H cells with or without overexpression of EGFR. Scale bar, 500 μm. Data presented as mean ± SD (three technical replicates). (J) Representative images and quantification of invasion in METTL1‐knockdown MHCC97H cells with or without overexpression of EGFR. Scale bar, 500 μm. Data presented as mean ± SD (three technical replicates). *p < .05, **p < .01, ***p < .001 by Student's *t* test, one‐way ANOVA or the Mann–Whitney U test unless specified. All the in vitro assays were biologically repeated for three times. Abbreviations: NC, negative control; oeEGFR, overexpression of EGFR; oeLysCTT, overexpression of LysCTT; siM1, siMETTL1‐1

Furthermore, we performed rescue experiments to separately overexpress tRNA LysCTT or EGFR. We found that forced expression of tRNA LysCTT or EGFR could partially rescue cancer progression in siMETTL1 MHCC97H and Huh7 cells (Figure 5A–J and Figure S8A–D). In addition, tRNA LysCTT overexpression also partially rescued EGFR and Cyclin A2 expression in siMETTL1 MHCC97H and Huh7 cells (Figure 5A). Overall, we demonstrated that reduced m^7^G tRNA modification impairs the translation of certain oncogenic mRNAs, resulting in the suppression of the HCC progression.

### 3.5 WDR4 knockdown reduces m^7^G tRNA modification and suppresses HCC progression

Given that WDR4 is another important component of the catalyzing complex for m^7^G tRNA modification, we further examined the function of WDR4 depletion in HCC progression (Figure S9A). Consistent with the results in METTL1 knockdown cells, WDR4 depletion dramatically inhibited cell growth, clone formation, cell cycle progression, migration and invasion (Figures S8J–S9B), and significantly increased cell apoptosis (Figure S9K, L) in Huh7 and MHCC97H cells. Similar phenomena were observed in subcutaneous xenograft model that WDR4 knockdown inhibited tumour growth, reduced tumour size and weight in vivo (Figure S9M‐O). In addition, our polyribosome‐associated mRNA‐qPCR assay confirmed the decreased TEs of Cyclin A2, EGFR and VEGFA mRNAs in WDR4‐knockdown cells (Figure S10A, B). And the decreased TE resulted in the reduced Cyclin A2, EGFR and VEGFA protein expression levels (Figure S10C, D). Taken together, WDR4 depletion reduces m^7^G tRNA modification and leads to less malignant tumour behaviour in HCC cells, further supporting the importance of m^7^G tRNA modification in HCC progression.

### 3.6 Liver‐specific knockout of Mettl1 inhibits hepatocarcinogenesis in vivo

**FIGURE 6:**
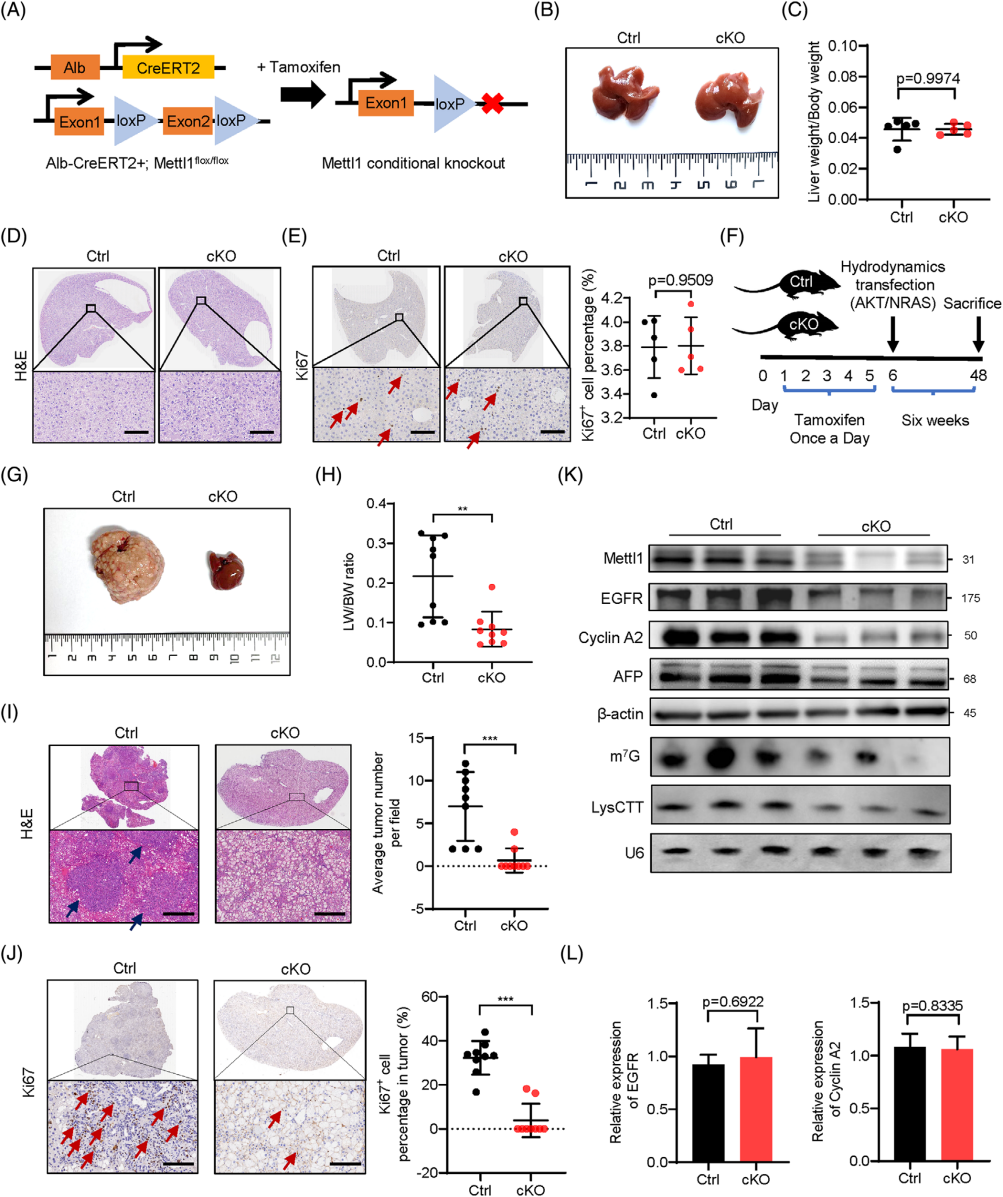
Liver‐specific knockout of Mettl1 inhibits HCC tumourigenesis in vivo. (A) The schematic diagram of the construction of Mettl1 conditional liver‐specific knockout mice. (B) Representative image of livers harvested from Mettl1‐cKO and control group. (C) Comparison of ratio of liver weight to body weight between Mettl1‐cKO and control group. Data presented as mean ± SD (*n* = 5). (D) Representative images of H&E staining of control and Mettl1‐cKO mouse livers. Scale bar: 100 μm. (E) IHC staining of Ki‐67 in control and Mettl1‐cKO mouse livers. Arrows point to Ki67‐positive cells. Scale bar:100 μm. Data presented as mean ± SD (*n* = 5). (F) General view of hydrodynamics transfection experiment in Mettl1 conditional knockout mice and control. (G) Representative image of livers harvested from Mettl1‐cKO and control group. (H) Comparison of tumour burden between Mettl1‐cKO and control group by ratio of liver weight to body weight (LW/BW). Data presented as mean ± SD (*n* = 9). (I) Representative images of H&E staining of control and Mettl1‐cKO mouse livers. Arrows point to tumour lesions. Scale bar: 250 μm. The number of tumour foci in these mouse livers was evaluated. Data presented as mean ± SD (*n* = 9). (J) Left panel, representative images of IHC staining of Ki‐67. Arrows point to Ki67‐positive cells. Scale bar:100 μm. Right panel, the statistical analyses. Data presented as mean ± SD (*n* = 9). (K) Western blot of Mettl1, EGFR, Cyclin A2 and AFP in tumour tissues from control group and Mettl1‐cKO group. β‐Actin was used as a loading control. Downregulation of m^7^G tRNA modification and LysCTT in Mettl1‐cKO group was confirmed by northwestern blot and northern blot. U6 was used as a loading control. (L) qRT‐PCR analysis of Cyclin A2 and EGFR mRNA levels in tumour tissues from control group and Mettl1‐cKO group. Data presented as mean ± SD (*n* = 3). *p < .05, **p < .01, ***p < .001 by Student's *t* test, one‐way ANOVA or the Mann–Whitney U test unless specified. All the in vitro assays were biologically repeated for three times. Abbreviations: cKO, conditional knockout; Ctrl, control

To further validate the role of m^7^G tRNA modification in HCC tumourigenesis in vivo, we established tamoxifen‐induced liver‐specific Mettl1 knockout mouse model (Alb‐CreERT2; Mettl1^flox/flox^, cKO) (Figure 6A). We first induced Mettl1 knockout with tamoxifen and found that Mettl1 condition knockout had little effect on liver weight, liver morphology and the proliferation of liver cells (Figure 6B–E). We then used a well‐established HCC model to induce HCC tumourigenesis in control (Ctrl) and cKO mice through hydrodynamic transfection of myr‐AKT1 and N‐RasV12 plasmids^33^ (Figure 6F). Six weeks after transfection, the control mice showed dramatic liver enlargement and tumour burden, while the Mettl1‐cKO group mice had smaller liver size (Figure 6G) and decreased tumour burden (Figure 6H). In addition, H&E staining showed that there were significantly decreased tumour lesions in Mettl1‐cKO mice (Figure 6I). Furthermore, IHC staining showed that tumours in the Mettl1‐cKO mice had lower percentage of Ki67‐positive cells and decreased intensity of HCC marker AFP (Figure 6J and Figure S11A). As revealed by western, northern and northwestern blot, AFP, EGFR and Cyclin A2 protein levels, and tRNA LysCTT expression level were downregulated in the liver tissues of Mettl1‐cKO mice (Figure 6K). However, there was little change in the mRNA levels of EGFR and Cyclin A2 (Figure 6L). Comparisons of m^7^G tRNA modification and expression profile between mouse and human liver cancer revealed good correlation of m^7^G tRNA modification and expression between mouse liver cancer and MHCC97H cells (Figure S12A, B), suggesting similar regulatory mechanisms of METTL1 in HCC progression between mouse and human. Overall, our results demonstrated the important role of METTL1 and m^7^G tRNA modification in hepatocarcinogenesis in vivo.

### 3.7 Overexpression of METTL1 promotes HCC cell growth, migration and invasion in vitro

To further confirm METTL1's function in HCC, we next overexpressed METTL1 in SNU‐449 and the immortalized normal liver cell line THLE‐2. Lentiviral transduction of wild‐type METTL1 and the METTL1 mutant (with defected enzymatic activity of METTL1) successfully induced METTL1 overexpression in SNU‐449 cells (Figure S13A, C). Our data revealed that overexpression of wild‐type METTL1 increased cell growth in both SNU‐449 and THLE‐2 (Figure S13B, D). Moreover, overexpression of METTL1 facilitates the migration and invasion of SNU‐449 cells (Figure S13E, F), while the METTL1 catalytic inactive mutant fails to promote cell growth and HCC progression (Figure S13A‐F). Furthermore, our polyribosome‐associated mRNA‐qPCR assay revealed that overexpression of wild‐type METTL1, but not catalytic inactive mutant, increased the TEs and protein levels of Cyclin A2 and EGFR in SNU‐449 cells (Figure S13G, H). In summary, our gain‐of‐function studies supported the important function of that m^7^G tRNA modification in promoting HCC progression in vitro.

### 3.8 Mettl1 knockin in mouse promotes in vivo hepatocarcinogenesis

**FIGURE 7:**
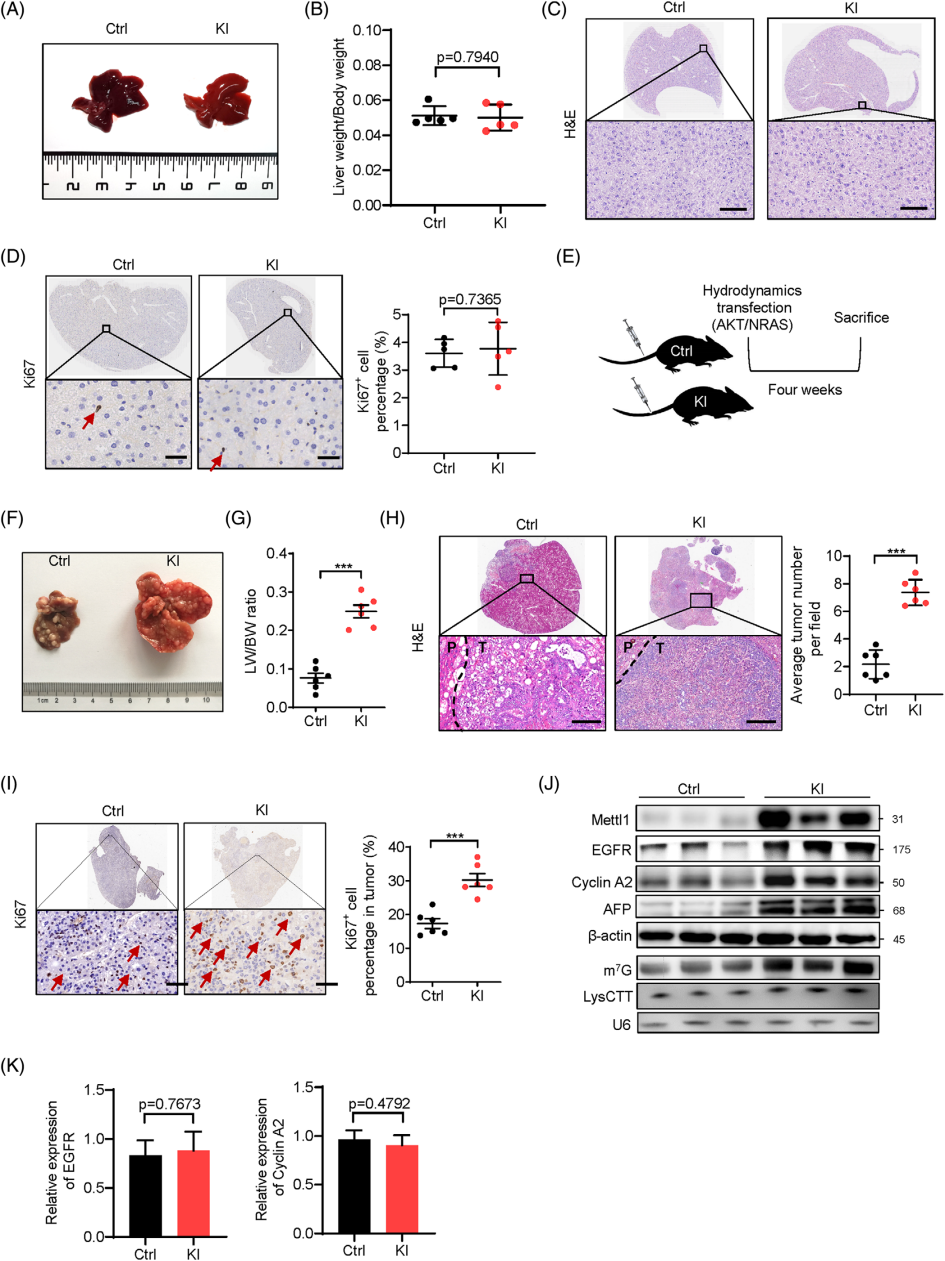
Overexpression of METTL1 promotes HCC progression in vivo. (A) Representative image of livers harvested from Mettl1‐KI and control group. (B) Comparison of ratio of liver weight to body weight between Mettl1‐KI and control group. Data presented as mean ± SD (*n* = 5). (C) Representative images of H&E staining of control and Mettl1‐KI mouse livers. Scale bar: 100 μm. (D) IHC staining of Ki‐67 in control and Mettl1‐KI mouse livers. Arrows point to Ki67‐positive cells. Scale bar: 50 μm. Data presented as mean ± SD (*n* = 5). (E) General view of hydrodynamics transfection experiment. AKT and NRAS plasmids along with SB transposase were injected into control and Mettl1 knockin mice (Mettl1‐KI mice). The mice were sacrificed after 4 weeks. *n* = 6. (F) Representative image of livers harvested from Mettl1‐KI and control group. (G) Comparison of tumourigenic capacity between Mettl1‐KI and control group by ratio of liver weight to body weight (LW/BW). Data presented as mean ± SD (*n* = 6). (H) Representative images of H&E staining of control and Mettl1‐KI mouse livers. Scale bar: 100 μm. The number of tumour foci in these mouse livers was evaluated. P, peri‐tumour; T, tumour. Data presented as mean ± SD (*n* = 6). (I) IHC staining of Ki‐67. Scale bar: 50 μm. Arrows point to Ki67‐positive cells. Data presented as mean ± SD (*n* = 6). (J) Western blot of Mettl1, EGFR, Cyclin A2 and AFP in tumour tissues from control group and Mettl1‐KI group. β‐Actin was used as a loading control. Upregulation of m^7^G tRNA modification and LysCTT in Mettl1‐KI group was confirmed by northwestern blot and northern blot. U6 was used as a loading control. (K) qRT‐PCR analysis of Cyclin A2 and EGFR mRNA levels in tumour tissues from control group and Mettl1‐KI group. Data presented as mean ± SD (*n* = 3). *p < .05, **p < .01, ***p < .001 by Student's *t* test, one‐way ANOVA or the Mann–Whitney U test unless specified. All the in vitro assays were biologically repeated for three times. Abbreviations: Ctrl, control; KI, knockin

We next established a Mettl1 knockin mouse model (Mettl1‐KI) to study the role of m^7^G tRNA modification in HCC tumourigenesis in vivo. Knockin of Mettl1 in mice cannot induce hepatocarcinogenesis in liver (Figure 7A–D), suggesting that Mettl1 overexpression is not strong enough to drive tumour formation. Then, we induced HCC tumourigenesis in Mettl1‐KI and control mice through hydrodynamic transfection (Figure 7E). Co‐expression of myr‐AKT1 and N‐RasV12 resulted in obvious liver enlargement and tumour burden within 4 weeks after injection in Mettl1‐KI group, resulting in significantly upregulation of liver weight to body weight ratio (Figure 7F, G). In addition, Mettl1‐KI mice had increased tumour densities than the control mice (Figure 7H). Moreover, histological analysis revealed that tumours in the Mettl1‐KI mice have higher percentage of Ki67‐positive cells and increased intensity of HCC marker AFP (Figure 7I and Figure S11B), suggesting the higher proliferative activity in the tumours from the KI mice. Our data further showed that the levels of Mettl1, EGFR, Cyclin A2, AFP, m^7^G tRNA modification and tRNA LysCTT are upregulated in the liver tissues of Mettl1‐KI mice (Figure 7J), while there is not significant difference in the mRNA expression levels of EGFR and Cyclin A2 (Figure 7K). Consistent with these results, Cyclin A2 and EGFR protein levels but not their mRNA levels are elevated in human HCC tissues compared to the peritumour tissues (Figure S14A, B). Taken together, our data uncovered the critical role of Mettl1‐mediated m^7^G tRNA modification in promoting hepatocarcinogenesis in vivo.

## 4 DISCUSSION

RNA modifications modulate many aspects of RNA metabolism and influence mRNA translation, and have emerged as important regulators in cancers, including HCC.^34^ m^7^G tRNA modification is a non‐essential tRNA modification in yeast. Depletion of m^7^G tRNA modification has no effect on yeast growth under normal condition, but causes growth deficiency under high temperature.^31^, ^35^ Similarly, a recent study reported that m^7^G tRNA modification regulates hydrogen peroxide response through translational regulation of Phe‐ and Asp‐ enriched genes in *Pseudomonas aeruginosa*.^36^ These findings suggest that m^7^G tRNA modification has little effect under normal condition but plays an important role in response to environmental stress in prokaryotes and lower eukaryotes. Our previous studies^12^, ^26^ and other recent studies^37^, ^38^ revealed that the m^7^G tRNA modification landscape in mammals is more complex than in the yeast. Knockout of Mettl1 or Wdr4 in mouse embryonic stem cells reduces the translation of neural genes and, therefore, impairs stem cells self‐renewal and neural differentiation.^12^ However, the knowledge about overactive m^7^G tRNA modification in HCC is limited. In this study, using clinical patient samples, HCC cell culture models, xenograft model, especially the Mettl1 knockin and conditional knockout mouse models, we demonstrated strong evidence supporting the critical function of METTL1/WDR4‐mediated m^7^G tRNA modifications in promoting HCC tumourigenesis and progression in vitro and in vivo, highlighting the important physiological functions of overactive m^7^G tRNA modification in HCC. Due to its important function of regulating tumour behaviour, aberrant m^7^G modification in HCC has great potential to be a target for the treatment of HCC patients.

It is well known that cancer cells have altered translation control to facilitate specific cancer cell behaviours.^39^ The cancer cells exhibit overactive translation activity that promotes the translation of a subset of oncogenic mRNAs.^39^ Various mechanisms could lead to selective translation enhancement. For example, the mTOR signalling pathway selectively enhances the translation of mRNAs with 5′terminal oligopyrimidine motifs.^40^ In addition, the m^6^A modifications on mRNAs can also promote specific oncogenic mRNA translation in cancers.^41^, ^42^, ^43^ Here, we found that METTL1‐mediated m^7^G tRNA modification and m^7^G codons regulate mRNA translation through mRNA codon frequency‐dependent manner, which demonstrates a novel mechanism of selective translation leading to malignant tumour behaviour. Overall, our data uncover a new molecular mechanism underlying the tRNA modification‐mediated mRNA translation enhancement in HCC.

The tRNAs are the most heavily modified RNA species in the cells. Depending on the modified positions on the tRNAs, the tRNA modifications could play different function in the regulation of tRNA processing and functions. Modifications on the anti‐codon positions could regulate the codon recognition functions of tRNAs, while modification on other positions could be essential for the appropriate tRNA structure and stability.^7^ Currently, the research on how these complex and dynamic tRNA modifications in the regulation of cancers is lagging behind.^44^ The recent development of the high‐throughput sequencing strategies for tRNA modification and expression profiling could facilitate the research on the function and mechanisms of tRNA modification in cancers,^25^, ^26^, ^27^ and, therefore, identify novel therapeutic targets for cancer therapy.

In summary, our study reveals the essential role of m^7^G tRNA modification in hepatocarcinogenesis, and uncovers new insights into gene expression regulation mediated by m^7^G tRNA modification and codon preference, providing strong evidence to support the important physiological function of misregulated m^7^G tRNA modifications in HCC progression. Therefore, targeting m^7^G tRNA modification is a promising strategy for the treatment of HCC patients.

## CONFLICT OF INTEREST

The authors have declared that no conflict of interest exists.

## Supporting information

SUPPORTING INFORMATION

## Data Availability

The polyribosome associated mRNA sequencing data and tRNA m7G reduction and cleavage sequencing data that support the findings of this study are openly available in [NCBI Gene Expression Omnibus (GEO)] at [https://www.ncbi.nlm.nih.gov/geo/query/acc.cgi?acc=GSE174492], reference number [GSE174492]. The other data that support the findings of this study are available from the corresponding author upon reasonable request.
