# Supplementary material for: METTL1 promotes hepatocarcinogenesis via m7G tRNA modification‐dependent translation control
Source: Clin Transl Med. 2021 Dec 12;11(12):e661. doi: 10.1002/ctm2.661 (PMC8666584; doi:10.1002/ctm2.661)
Supplement: Supplementary file 1 — SUPPORTING INFORMATION [file CTM2-11-e661-s001.docx]

**METTL1 promotes** **hepatocarcinogenesis via m^7^G tRNA modification dependent translation control**

Zhihang Chen^1, †^, Wanjie Zhu^2, †^, Shenghua Zhu^2, †^, Kaiyu Sun^3^, Junbin Liao^1^, Haining Liu^1^, Zihao Dai^1^, Hui Han^4^, Xuxin Ren^5^, Qingxia Yang^5^, Siyi Zheng^4,5^, Baogang Peng^1^, Sui Peng^2,5^, Ming Kuang^1,5,6,*^, Shuibin Lin^4,5,7,*^

**Contents**

**Table S1.** Baseline clinical characteristics of the hepatocellular carcinoma patients

**Table S2.** siRNA primer and probe sequences

**Table S3.** Primary antibodies

**Figure S1.** Analysis of TCGA-LIHC data.

**Figure S2.** Interaction between METTL1 and WDR4 *in vitro*.

**Figure S3.** Inhibition of METTL1 impairs HCC progression of Huh7 cells.

**Figure S4.** Cleavage scores of the 17 identified tRNAs.

**Figure S5.** Overexpression of METTL1 upregulates m^7^G-modified tRNA methylation level and expression.

**Figure S6.** Knockdown of WDR4 reduces m^7^G modification and suppresses global mRNA translation.

**Figure S7.** Decreased translation of Cyclin A2 induces G2/M cell cycle arrest.

**Figure S8.** Overexpression of EGFR partially rescued cancer phenotypes in knockdown Huh7 cells.

**Figure S9.** Inhibition of WDR4 suppresses the HCC progression.

**Figure S10.** WDR4 depletion inhibits translation of Cyclin A2 and EGFR.

**Figure S11.** Immunochemistry (IHC) staining of AFP.

**Figure S12.** Correlation of tRNA m^7^G methylome between mouse liver cancer and MHCC97H cells.

**Figure S13.** Overexpression of METTL1 promotes HCC cells progression and translation of Cyclin A2 and EGFR.

**Figure S14.** Expression levels of EGFR, Cyclin A2 and tRNA-LysCTT in HCC clinical samples.

**Table S1. Baseline clinical characteristics of the hepatocellular carcinoma patients**

|  | Total (n=57) |
| --- | --- |
| Age, yrs, mean±SD | 52.4 (11.5) |
| Gender |  |
| Male | 47 (82.5%) |
| Female | 10 (17.5%) |
| HBsAg |  |
| Negative | 4 (7.0%) |
| Positive | 53 (93.0%) |
| AFP, μg/L | 134.7 (25.4,722.7) |
| Tumor number |  |
| 1 | 57 (100.0%) |
| Tumor size, cm | 3.9 (2.9,4.3) |
| BCLC stage |  |
| 0 | 5 (8.8%) |
| A | 48 (84.2%) |
| C | 4 (7.0%) |
| Child-Pugh class |  |
| A | 54 (94.7%) |
| B | 3 (5.3%) |

Notes: Continuous variables are presented as median (inter-quartile range, IQR) unless noted otherwise. Categorical variables are presented as n (%).

**Table S2.** siRNA primer and probe sequences

| siMETTL1-1 | GATGACCCAAAGGATAAGAAA |
| --- | --- |
| siMETTL1-2 | GGATGTGCACTCATTTCGA |
| Human METTL1 forward primer | 5’- GGCAACGTGCTCACTCCAA-3 |
| Human METTL1 reverse primer | 5’-CACAGCCTATGTCTGCAAACT-3’ |
| Human WDR4 forward primer | 5’- ACAGCCCTGACTTTCATAGCC -3’ |
| Human WDR4 reverse primer | 5’- TCACAGCCACATCTAACAGCATA -3’ |
| Human Cyclin A2 forward primer | 5’- TGTCACCGTTCCTCCTTGG -3’ |
| Human Cyclin A2 reverse primer | 5’- GGGCATCTTCACGCTCTATTT -3’ |
| Human EGFR forward primer | 5’- GCTCTACAACCCCACCACG -3’ |
| Human EGFR reverse primer | 5’- GCCCTTCGCACTTCTTACACT -3’ |
| Human RPS10 forward primer | 5’- GTATCCAGTATCTCCGTGATT -3’ |
| Human RPS10 reverse primer | 5’- CTCCGTCTGTAGGTATCTCT -3’ |
| Human β-actin forward primer | 5’- TTGCTGACAGGATGCAGAAG-3’ |
| Human β-actin reverse primer | 5’- ACTCCTGCTTGCTGATCCACAT-3’ |
| Mouse EGFR forward primer | 5’- CACAAGTAACAGGCTCAC -3’ |
| Mouse EGFR reverse primer | 5’- GTTGGACAGGATGGCTAA -3’ |
| Mouse Cyclin A2 forward primer | 5’- AGCCAGACATCACTAACAG -3’ |
| Mouse Cyclin A2 forward primer | 5’- GGAGGAGAGGAATCTATCAAT -3’ |
| Mouse β-actin forward primer | 5’- GATCTGGCACCACACCTTCT -3’ |
| Mouse β-actin reverse primer | 5’- GGGGTGTTGAAGGTCTCAAA -3’ |
| Human tRNA-AlaAGC forward primer | 5’- GGGAATTAGCTCAAGTGGTAG -3’ |
| Human tRNA-AlaAGC reverse primer | 5’- GAGAATGCGGGCATCGAT -3’ |
| Human tRNA-LysCTT forward primer | 5’- AGCTCAGTCGGTAGAGCATCAG -3’ |
| Human tRNA-LysCTT reverse primer | 5’- CGAACAGGGACTTGAACCCTGG -3’ |
| Human tRNA-LysTTT forward primer | 5’- CGGATAGCTCAGTCGGTAG -3’ |
| Human tRNA-LysTTT reverse primer | 5’- CCCGAACAGGGACTTGAA -3’ |
| Human tRNA-MetCAT forward primer | 5’- CAGCGCGTCAGTCTCATA -3’ |
| Human tRNA-MetCAT reverse primer | 5’- CTCTCTGAGGCTCGAACTC -3’ |
| Human tRNA-PheGAA forward primer | 5’- GCCGAAATAGCTCAGTTG -3’ |
| Human tRNA-PheGAA reverse primer | 5’- GATCGAACCAGGGACCTT -3’ |
| Human tRNA-ValTAC forward primer | 5’- GGTTCCATAGTGTAGTGGTTAT -3’ |
| Human tRNA-ValTAC reverse primer | 5’- GCTTGAACCCAGGACCTT -3’ |
| Human U6 snoRNA forward primer | 5’- CGCTTCGGCAGCACATATAC -3’ |
| Human U6 snoRNA reverse primer | 5’- TTCACGAATTTGCGTGTCAT -3’ |
| U6 snoRNA | TGGAACGCTTCACGAATTTG |
| tRNA-LysCTT | CGACCCTGAGATTAAGAGTC |

**Table S3.** Primary antibodies

| Antibodies | Source | Identifier |
| --- | --- | --- |
| Anti-METTL1 | Proteintech | Cat#14994-1-AP |
| Anti-WDR4 | Abcam | Cat#ab169526 |
| Anti-7-methylguanosine (m^7^G) | MBL International | Cat#RN017M |
| Anti-Cyclin A2 | Cell Signaling Technology | Cat#4656 |
| Anti-EGFR | Proteintech | Cat# 66455-1-IG |
| Anti-VEGFA | Proteintech | Cat#19003-1-AP |
| Anti-p-Akt | Cell Signaling Technology | Cat#4060 |
| Anti-p-p44/42 MAPK | Cell Signaling Technology | Cat#4370 |
| Anti-IgG | Proteintech | Cat#B900610 |
| Anti-Ki67 | Abcam | Cat#ab15580 |
| Anti-AFP | Abcam | Cat#Ab46799 |
| Anti-puromycin | MilliporeSigma | Cat# MABE343 |
| Anti-β-actin | Proteintech | Cat#20536-1-AP |
| Anti-β-tubulin | Proteintech | Cat#10094-1-AP |
| Anti-GAPDH | Proteintech | Cat#10494-1-AP |
| Anti-Digoxigenin-AP Fab fragments | Roche | Cat#11093274910 |

**
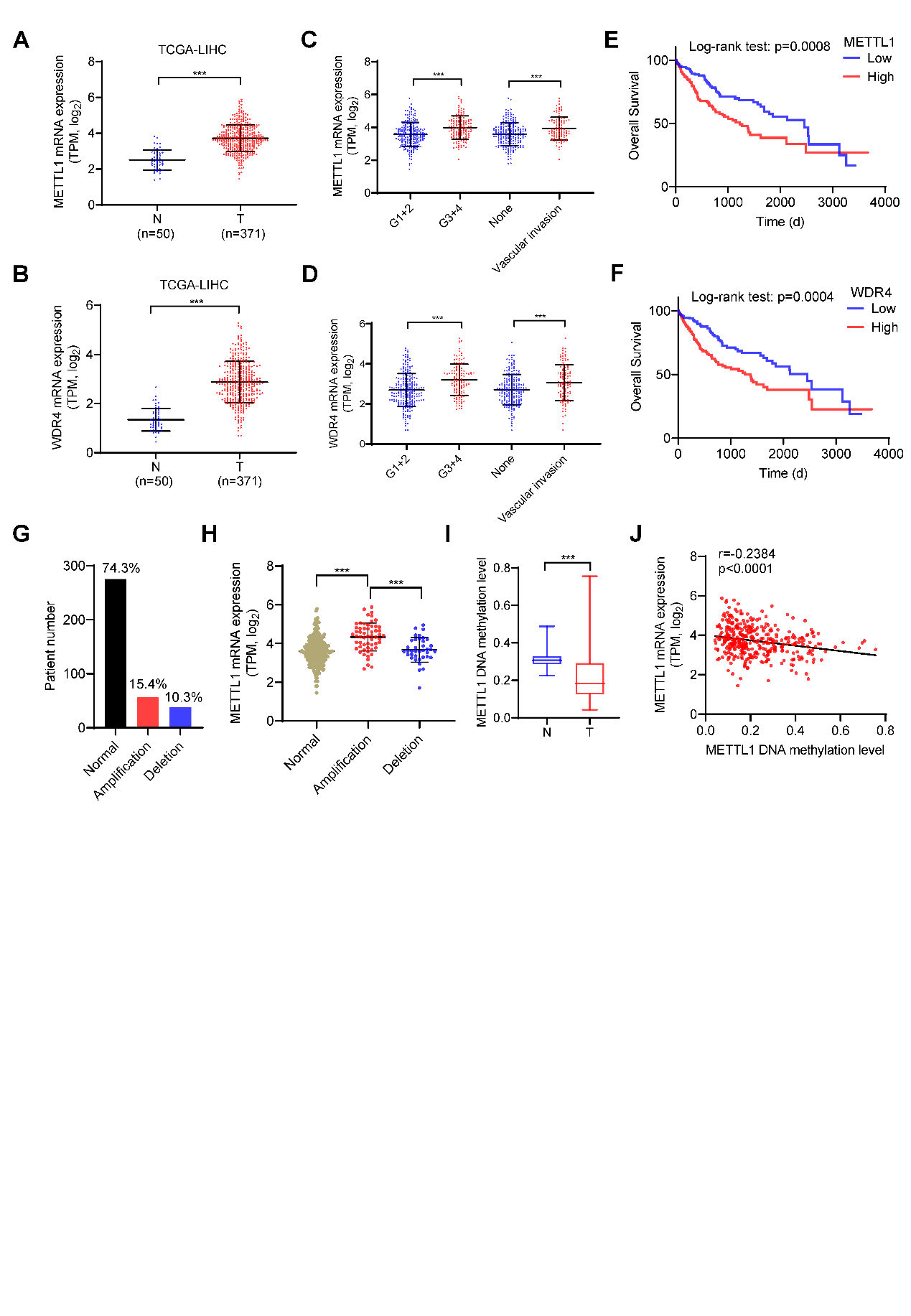
**

**Figure S1. Analysis of TCGA-LIHC data. (A-B)** METTL1 (**A**) and WDR4 (**B**) mRNA expression in TCGA LIHC dataset. Log_2_(TPM) was used for normalization. **(C-D)** METTL1 **(C)** and WDR4 **(D)** mRNA expression is correlated with tumor stage and vascular invasion. **(E-F)** Correlation between METTL1 **(E)** or WDR4 **(F)** mRNA expression and overall survival of HCC patients in TCGA dataset. Kaplan-Meier survival curve was plotted according to median METTLL and WDR4 mRNA expression. Log-rank test was used. **(G)** The number and percentage of copy amplification and loss in HCC patients of TCGA dataset (n=370). (**H**) Comparison of METTL1 mRNA expression among normal (n=275), amplification (n=57) and deletion (38) groups in TCGA dataset. (**I**) Comparison of METTL1 DNA methylation levels between normal liver and tumor tissues in TCGA LIHC dataset. (**J**) Correlation between METTL1 mRNA expression and METTL1 DNA methylation level. Pearson analysis was used. *p <0.05, **p < 0.01, ***p < 0.001 by Student’s t test or the Mann-Whitney U test unless specified. T, tumor tissue; N, normal tissue; TPM, transcripts per million.

**

**

**Figure S2. Interaction between METTL1 and WDR4 *in vitro*.** Anti-WDR4 antibodies were used for pull down METTL1. β-actin serves as a negative control.

**
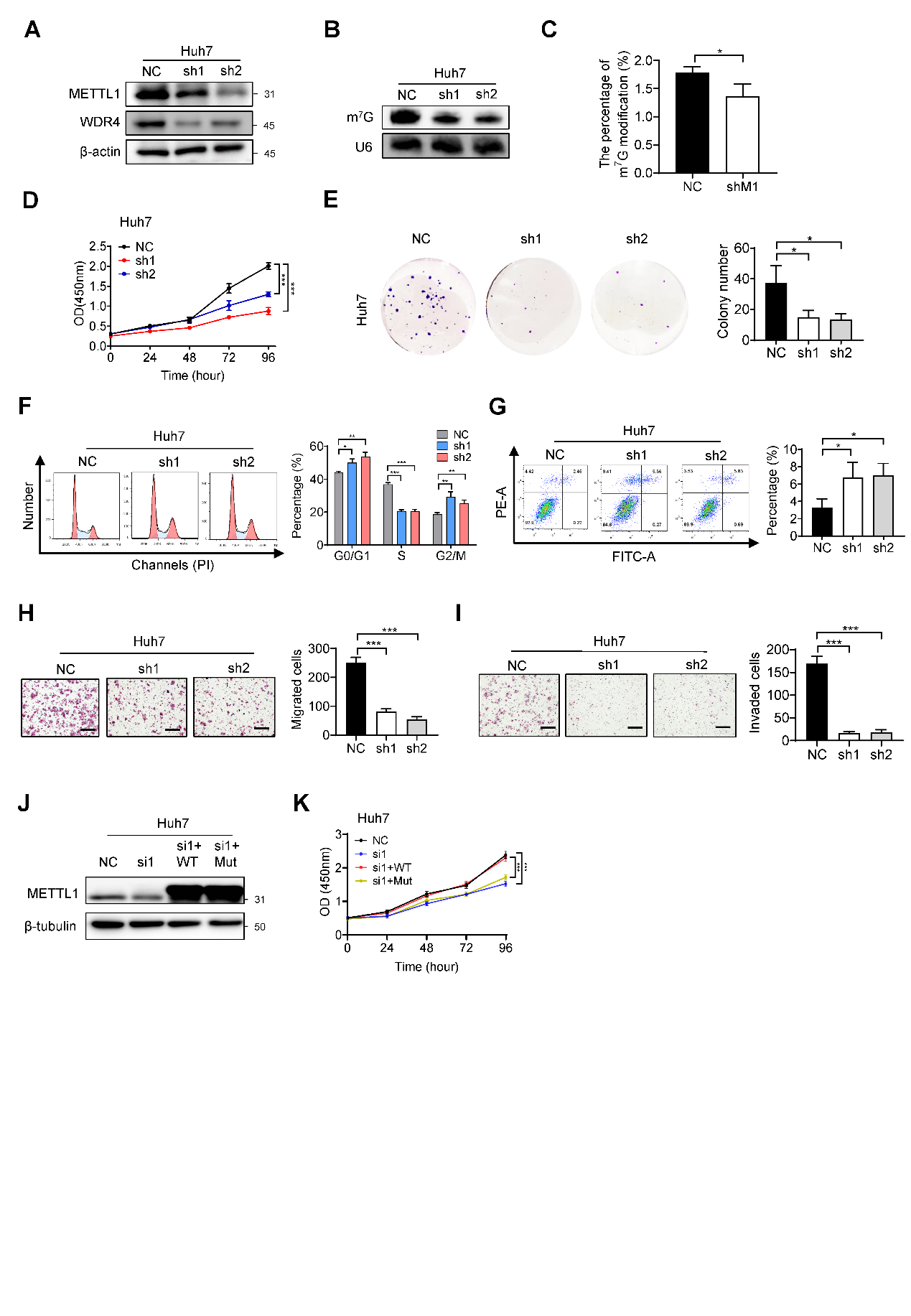
**

**Figure S3. Inhibition of METTL1 impairs HCC progression of Huh7 cells. (A)** The knockdown effect of METTL1 in Huh7 cells was confirmed by Western blot. **(B)** The downregulation of m^7^G tRNA modification was confirmed by northwestern blot. **(C)** Validation of the altered level of m^7^G tRNA modification upon METTL1 depletion in Huh7 cells by liquid chromatography-coupled mass spectrometry. shMETTL1-2 was used. Data presented as mean ± SD (n=3). **(D)** CCK-8 assay of METTL1 knockdown and control Huh7 cells. Data presented as mean ± SD (Six technical replicates). **(E)** Representative images and quantification of clone formation in METTL1 depleted and control Huh7 cells. Data presented as mean ± SD (Three technical replicates). **(F)** Cell cycle analysis and quantification of METTL1 depleted and control Huh7 cells. Data presented as mean ± SD (Three technical replicates). **(G)** Representative images and quantification of cell apoptosis assays in Huh7 cells with or without METTL1 knockdown. Data presented as mean ± SD (Three technical replicates). **(H)** Representative images and quantification of migration in METTL1 depleted and control Huh7 cells. Scale bar, 500 μm. Data presented as mean ± SD (Three technical replicates). **(I)** Representative images and quantification of invasion in METTL1 depleted and control Huh7 cells. Scale bar, 500 μm. Data presented as mean ± SD (Three technical replicates). **(J)** Validation of the rescue of METTL1 by Western blots in Huh7 cells. siMETTL1-1 was used. **(K)** CCK-8 assay of METTL1 knockdown Huh7 cells with rescue expression of wild type METTL1 or the mutant**.** *p <0.05, **p < 0.01, ***p < 0.001 by Student’s t test, one-way ANOVA or the Mann-Whitney U test unless specified. All the in vitro assays were biologically repeated for 3 times. sh1, shMETTL1-1; sh2, shMETTL1-2; NC, negative control; shM1, shMETTL1; si1, siMETTL1-1; WT, wild type METTL1; Mut, mutant METTL1.


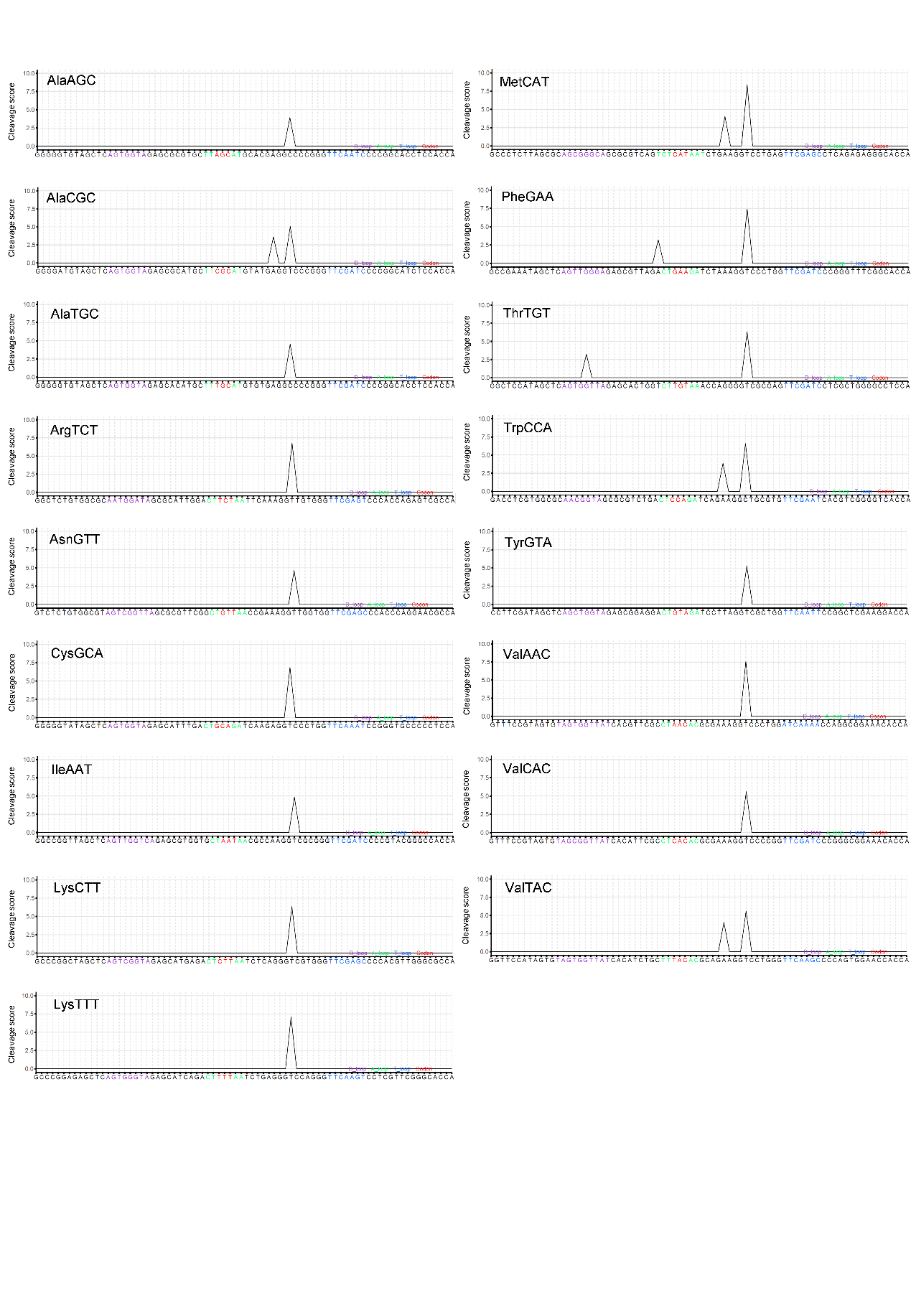


**Figure S4. Cleavage scores of the 17 identified tRNAs.** Pictures show a specific increase of cleavage score at the m^7^G sites.

**
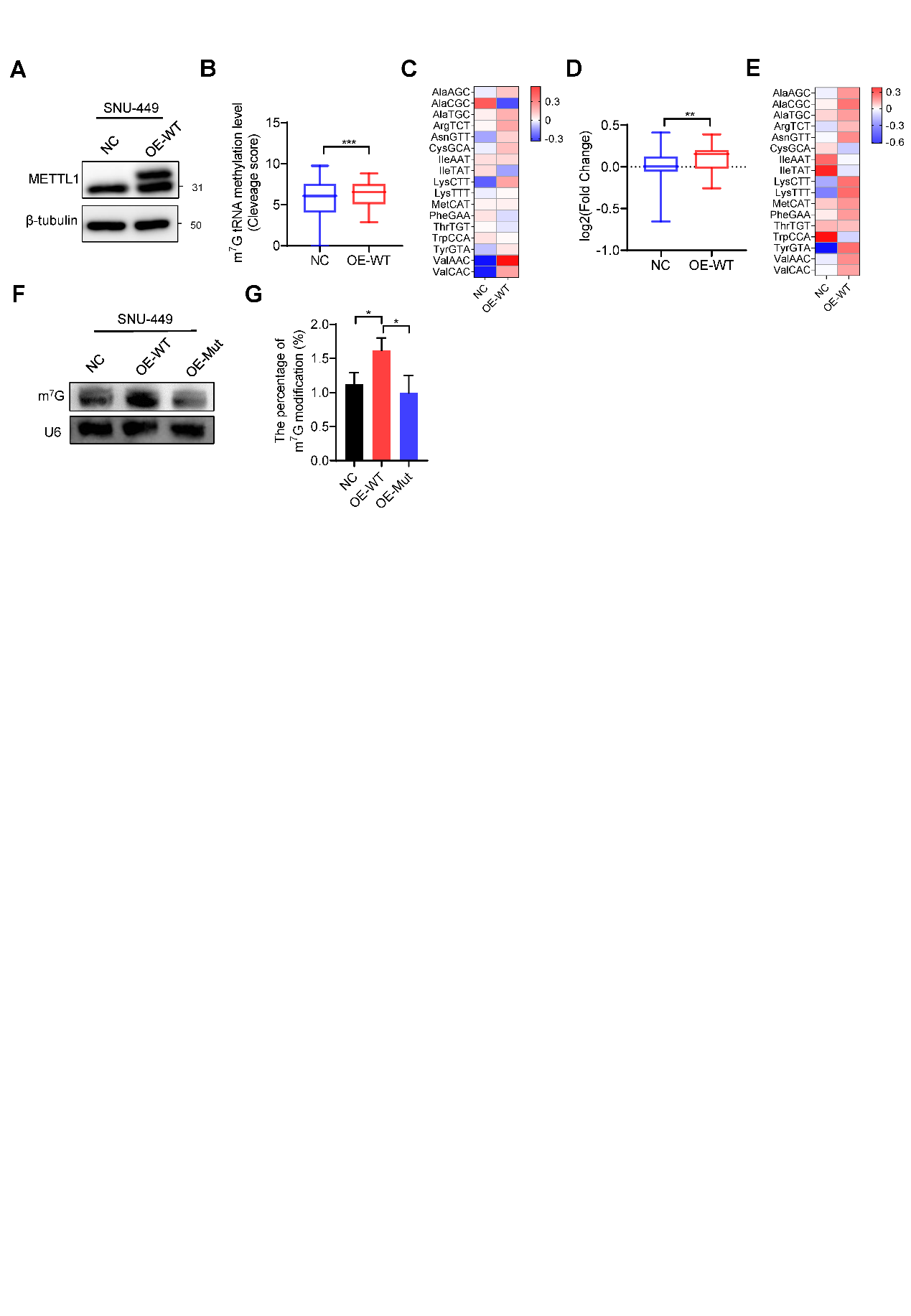
**

**Figure S5. Overexpression of METTL1 upregulates m^7^G-modified tRNA methylation level and expression. (A)** The overexpression of METTL1 in SNU-449 cells was confirmed by western blot. **(B)** Global m^7^G tRNA methylation level of SNU-449 cells with or without overexpression of METTL1 by TRAC-seq. Wilcoxon signed-rank test was used. **(C)** Methylation profile of the 17 m^7^G-modified tRNAs identified by TRAC-seq. The methylation level of each tRNA type was calculated from the combined cleavage score of all the tRNA genes belong to the same tRNA type. Methylation level of the indicated tRNA type was then normalized by its overall average level in both groups and transformed by log2. **(D)** Expression level of m^7^G-modified tRNAs between METTL1 overexpression and control group in SNU-449 cells. **(E)** Expression profile of the 17 m^7^G-modified tRNAs identified by TRAC-seq. The expression of each tRNA type was calculated from the combined expression of all the tRNA genes belong to the same tRNA type. Expression of the indicated tRNA type was then normalized by its overall average level in both groups and transformed by log2. **(F)** The overexpression effect of m^7^G tRNA modification was confirmed by northwestern blot. **(G)** Validation of the altered level of m^7^G tRNA modification upon METTL1 overexpression in SNU-449 cells by liquid chromatography-coupled mass spectrometry. NC, negative control; OE-WT, overexpression of wild type METTL1; OE-Mut, overexpression of mutant METTL1.


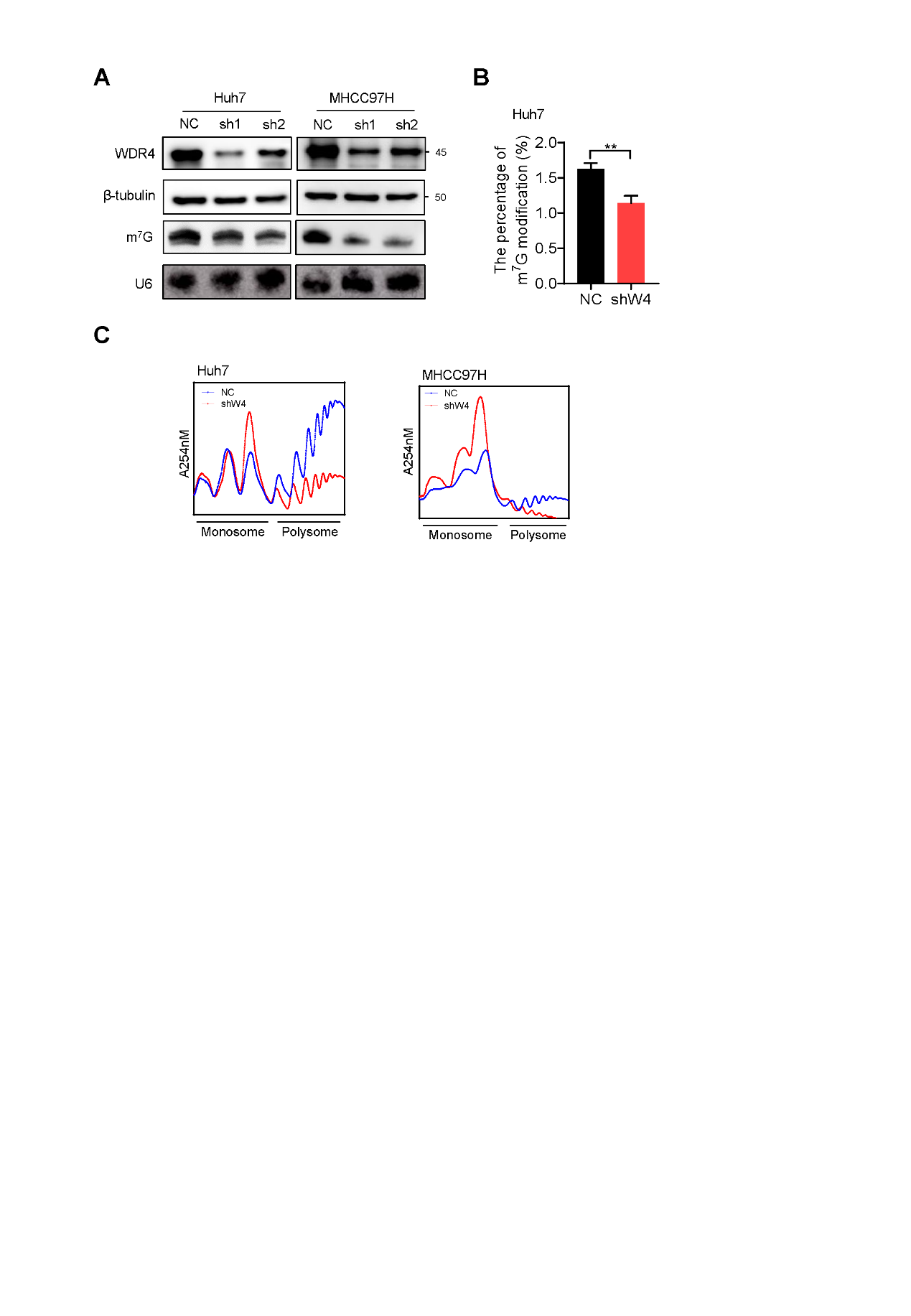


**Figure S6. Knockdown of WDR4 reduces m^7^G modification and suppresses global mRNA translation. (A)** The knockdown effects of WDR4 and m^7^G tRNA modification were confirmed by western blot and northwestern blot. **(B)** Validation of the altered level of m^7^G tRNA modification upon WDR4 deletion in Huh7 cells by liquid chromatography-coupled mass spectrometry. shWDR4-2 was used. **(C)** Polysome profiling of Huh7 and MHCC97H with or without WDR4 knockdown. shWDR4-2 was used in this experiment. *p <0.05, **p < 0.01, ***p < 0.001 by Student’s t test or the Mann-Whitney U test unless specified. sh1, shWDR4-1; sh2, shWDR4-2; shW4, shWDR4.


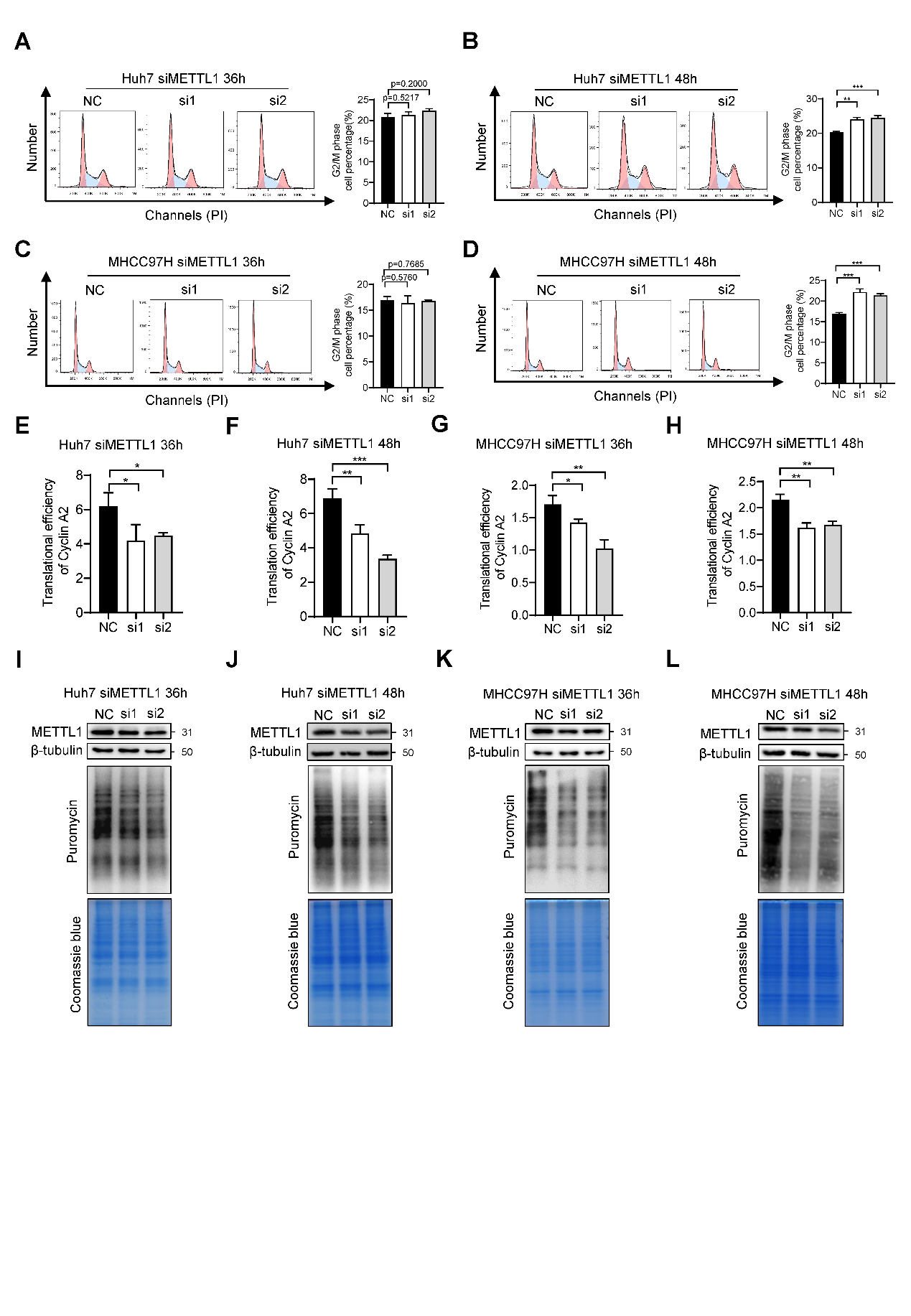


**Figure S7. Decreased translation of Cyclin A2 induces G2/M cell cycle arrest. (A-D)** Cell cycle analysis and quantification of Huh7 and MHCC97H cells at 36h and 48h after siRNA transfection. **(E-H)** Translation efficiency of Cyclin A2 of Huh7 and MHCC97H cells at 36h and 48h after siRNA transfection. **(I-L)** Global translation of Huh7 and MHCC97H cells at 36h and 48h after siRNA transfection. Coomassie brilliant blue staining of the gel was used as control. Data presented as mean ± SD. *p <0.05, **p < 0.01, ***p < 0.001 by Student’s t test or the Mann-Whitney U test. All the assays were biologically repeated for 3 times. si1, shMETTL1-1; si2, siMETTL1-2; NC, negative control.


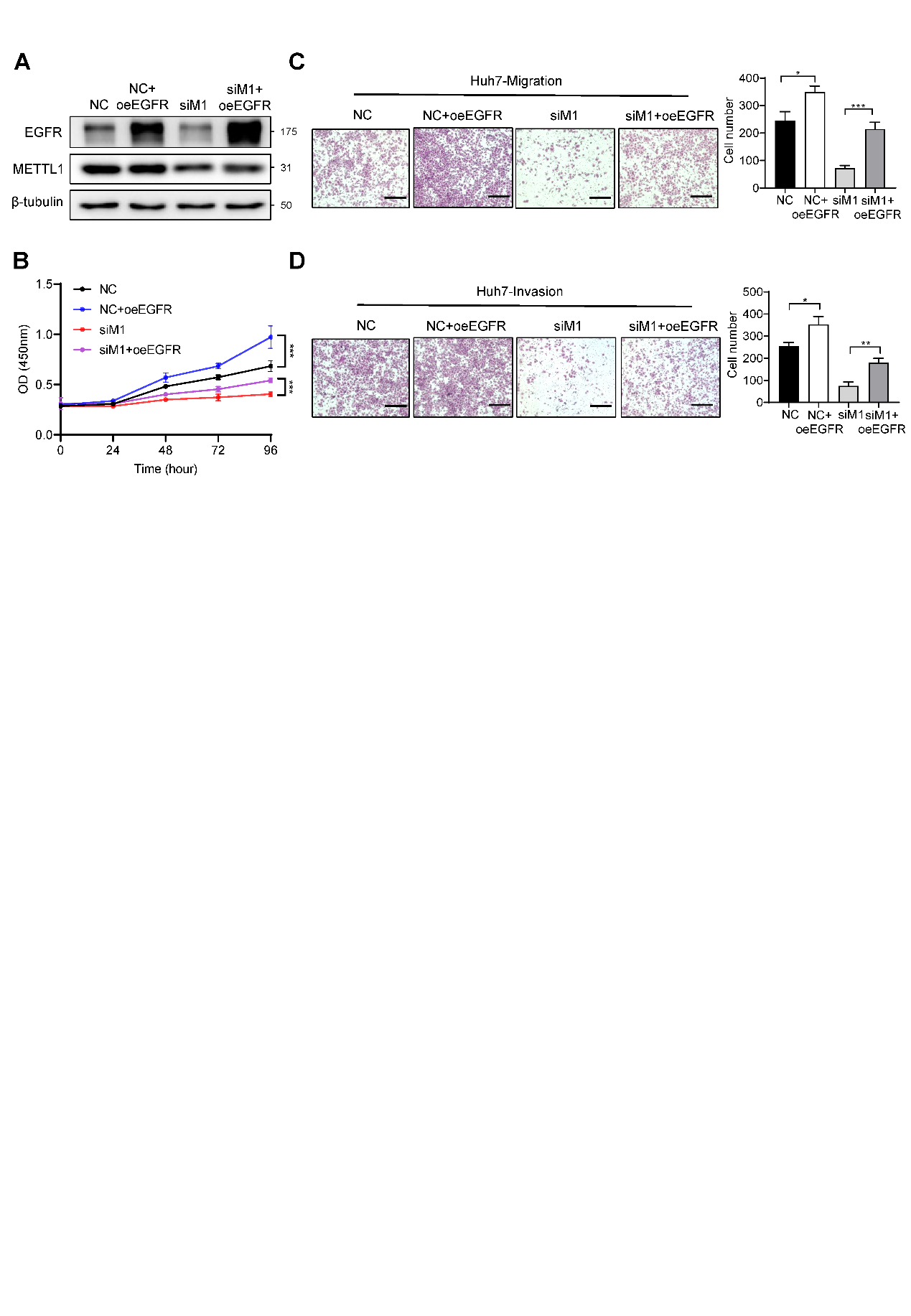


**Figure S8. Overexpression of EGFR partially rescued cancer phenotypes in knockdown Huh7 cells. (A)** Validation of the EGFR overexpression and METTL1 depletion by western blot in Huh7 cells. **(B)** CCK-8 assay of METTL1 knockdown Huh7 cells with or without overexpression of EGFR in Huh7 cells. Data presented as mean ± SD (Six technical replicates). **(C)** Representative images and quantification of migration in METTL1-knockdown Huh7 cells with or without overexpression of EGFR. Scale bar, 500 μm. Data presented as mean ± SD (Three technical replicates). **(D)** Representative images and quantification of invasion in METTL1-knockdown Huh7 cells with or without overexpression of EGFR. Scale bar, 500 μm. Data presented as mean ± SD (Three technical replicates). *p <0.05, **p < 0.01, ***p < 0.001 by Student’s t test, one-way ANOVA or the Mann-Whitney U test unless specified. All the in vitro assays were biologically repeated for 3 times. NC, negative control; siM1, siMETTL1-1; oeEGFR, overexpression of EGFR.


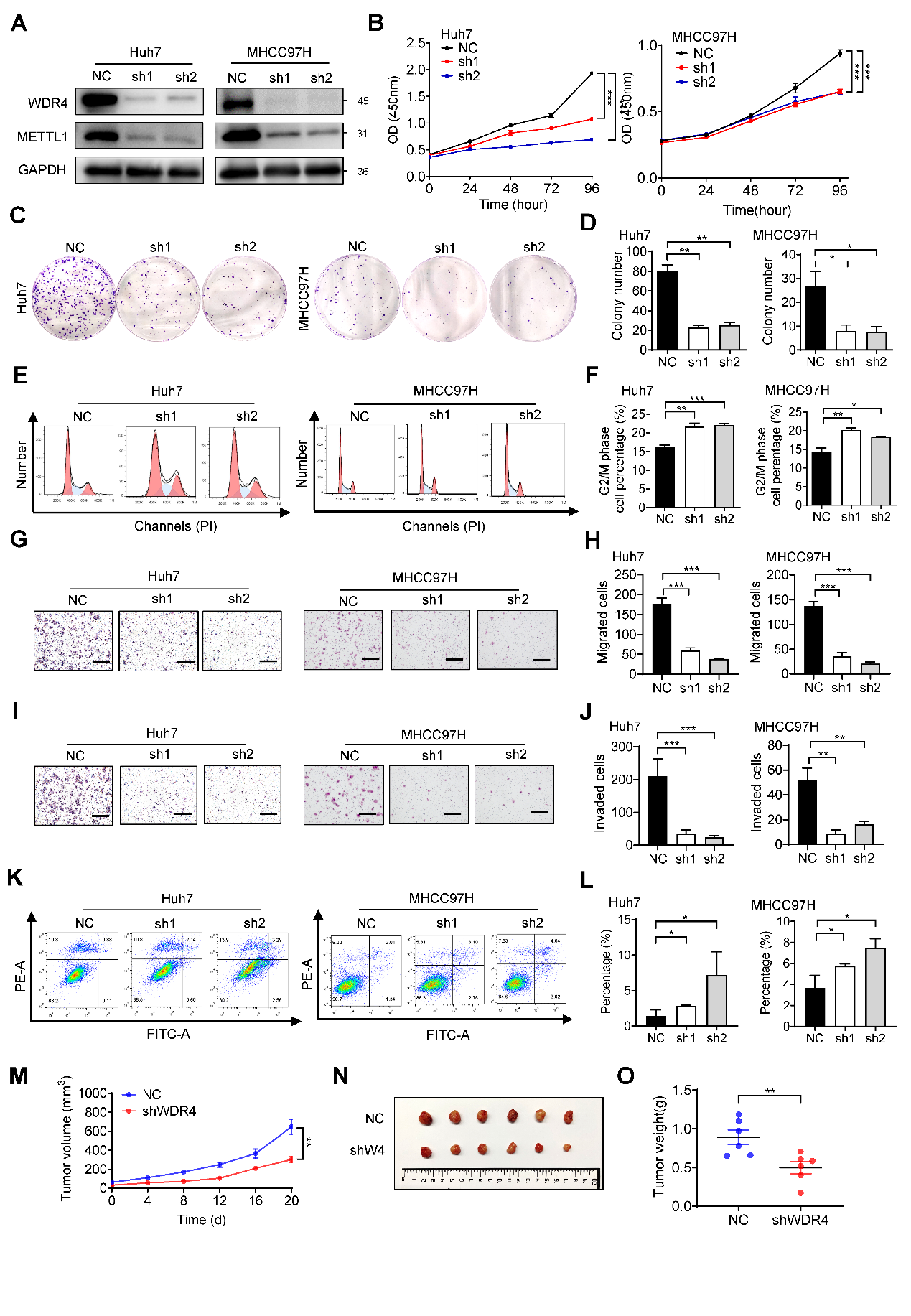


**Figure S9. Inhibition of WDR4 suppresses the HCC progression. (A)** Stable knockdown of WDR4 in MHCC97H and Huh7 cell lines by lentiviral shRNA sequences (shWDR4-1 and -2). The knockdown effect of WDR4 was confirmed by western blot. **(B)** CCK-8 assay of WDR4 depleted and control Huh7 and MHCC97H cells. Data presented as mean ± SD (Six technical replicates). **(C-D)** Representative images **(C)** and quantification **(D)** of clone formation in WDR4 depleted and control Huh7 and MHCC97H cells. Data presented as mean ± SD (Three technical replicates). **(E-F)** Cell cycle analysis **(E)** and quantification **(F)** of WDR4 depleted and control Huh7 and MHCC97H cells. Data presented as mean ± SD (Three technical replicates). **(G-H)** Representative images **(G)** and quantification **(H)** of migration in WDR4 depleted and control Huh7 and MHCC97H cells. Scale bar, 500 μm. Data presented as mean ± SD (Three technical replicates). **(I-J)** Representative images **(I)** and quantification **(J)** of invasion in WDR4 depleted and control Huh7 and MHCC97H cells. Scale bar, 500 μm. Data presented as mean ± SD (Three technical replicates). **(K-L)** Representative images **(K)** and quantification **(L)** of cell apoptosis assays in Huh7 or MHCC97H cells with or without WDR4 knockdown. Data presented as mean ± SD (Three technical replicates). **(M-O)** WDR4 knockdown significantly inhibited subcutaneous transplanted tumor growth. WDR4 depleted and control MHCC97H cells were implanted subcutaneously into nude mice. **(M)** Growth of subcutaneous transplanted tumors. Tumor size was measured every 4 days. **(N)** Overview of subcutaneous transplanted tumors. **(O)** Tumor weights at the time of sacrifice. Data presented as mean ± SEM (n=6). *p <0.05, **p < 0.01, ***p < 0.001 by Student’s t test, one-way ANOVA or the Mann-Whitney U test unless specified. All the in vitro assays were biologically repeated for 3 times. sh1, shWDR4-1; sh2, shWDR4-2; NC, negative control.





**Figure S10. WDR4 depletion inhibits translation of Cyclin A2 and EGFR. (A-B)** Relative expression and translation efficiency of Cyclin A2, EGFR and VEGFA mRNA in WDR4 depleted and control MHCC97H (**A**) and Huh7 (**B**) cells. β-actin was used as an internal control. Data presented as mean ± SD (Three technical replicates). **(C-D)** Western blot of Cyclin A2, EGFR, VEGFA, p-Akt and p-p44/42 MAPK in WDR4 depleted and control MHCC97H **(C)** and Huh7 **(D)** cells. *p <0.05, **p < 0.01, ***p < 0.001 by Student’s t test or the Mann-Whitney U test. All the assays were biologically repeated for 3 times. NC, negative control; sh1, shWDR4-1; sh2, shWDR4-2.

**
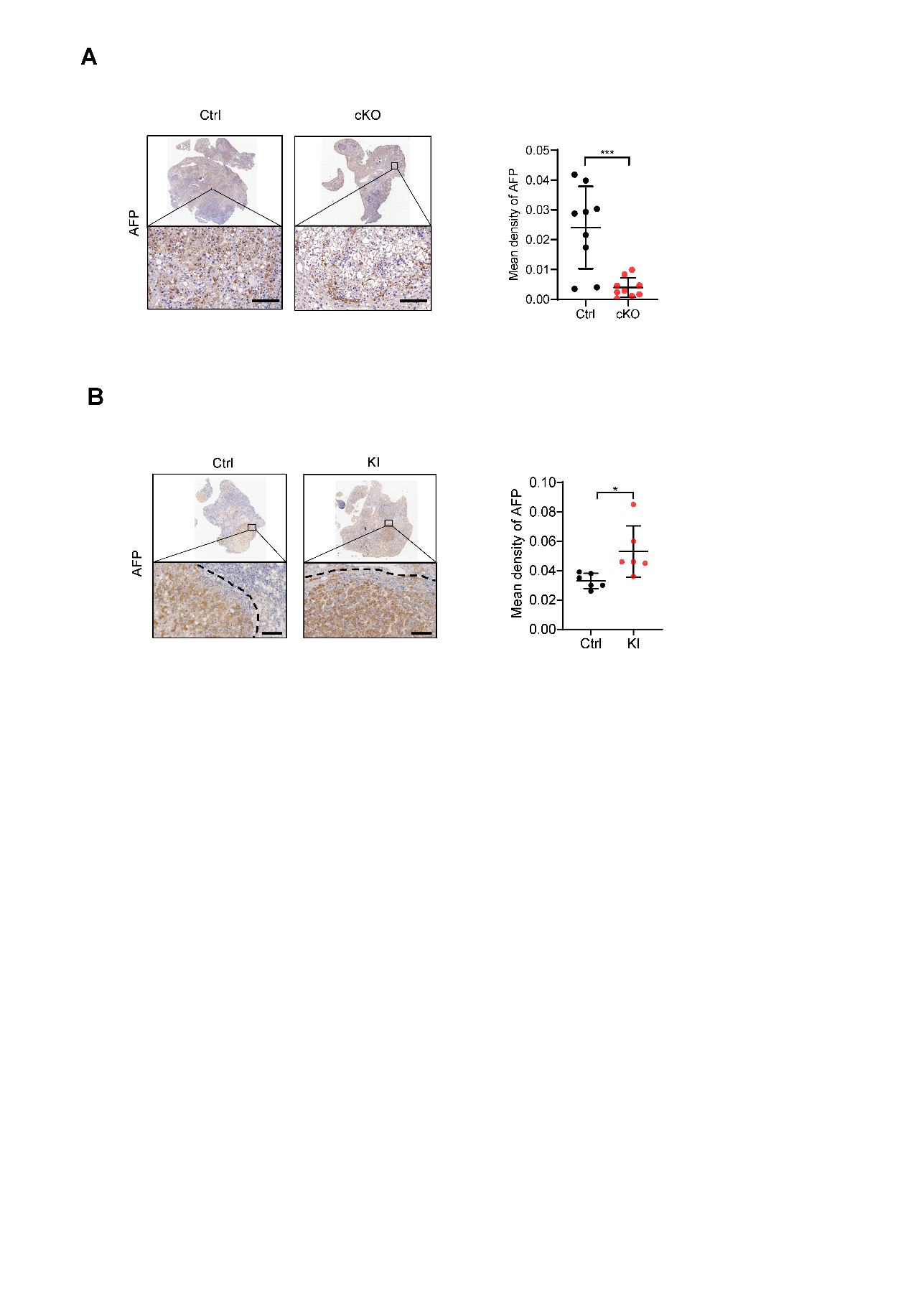
**

**Figure S11. Immunochemistry (IHC) staining of AFP.** (**A**) Representative images and quantification of IHC staining of AFP in Mettl1-cKO mice. Scale bar:100 μm. Data presented as mean ± SD (n=9). (B) Representative images and quantification of IHC staining of AFP in Mettl1-KI mice. Scale bar:50 μm. Data presented as mean ± SD (n=6). *p <0.05, **p < 0.01, ***p < 0.001 by Student’s t test, one-way ANOVA or the Mann-Whitney U test unless specified. Ctrl, control; cKO, conditional knockout; KI, knock-in.

**
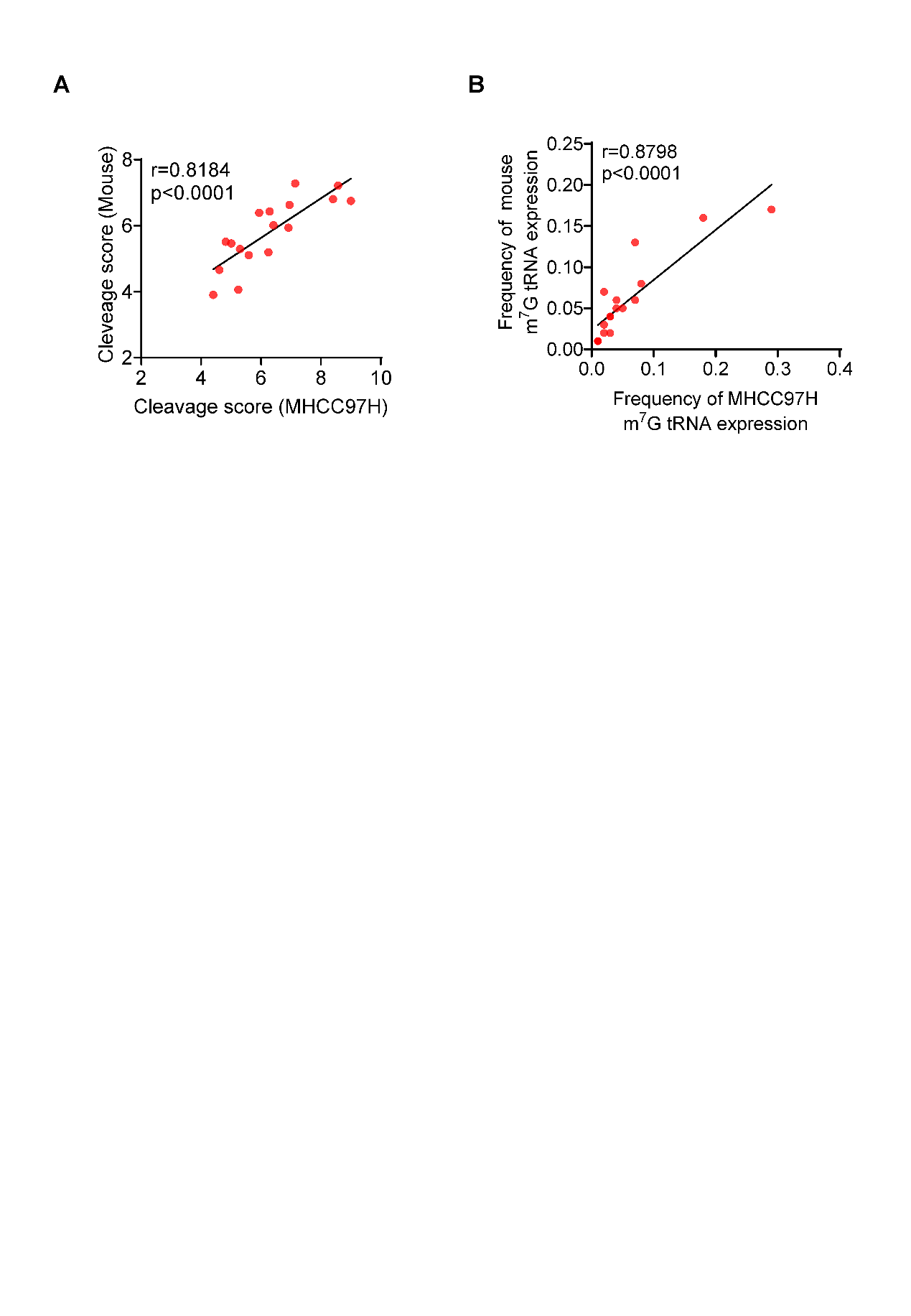
**

**Figure S12. Correlation of tRNA m^7^G methylome between mouse liver cancer and MHCC97H cells. (A)** Correlation of cleavage scores of m^7^G-modified tRNAs in mouse liver cancer and those in MHCC97H cells. Pearson correlation analysis was used. **(B)** Correlation of m^7^G-modified tRNAs expression in mouse liver cancer and those in MHCC97H cells. Pearson correlation analysis was used.

**
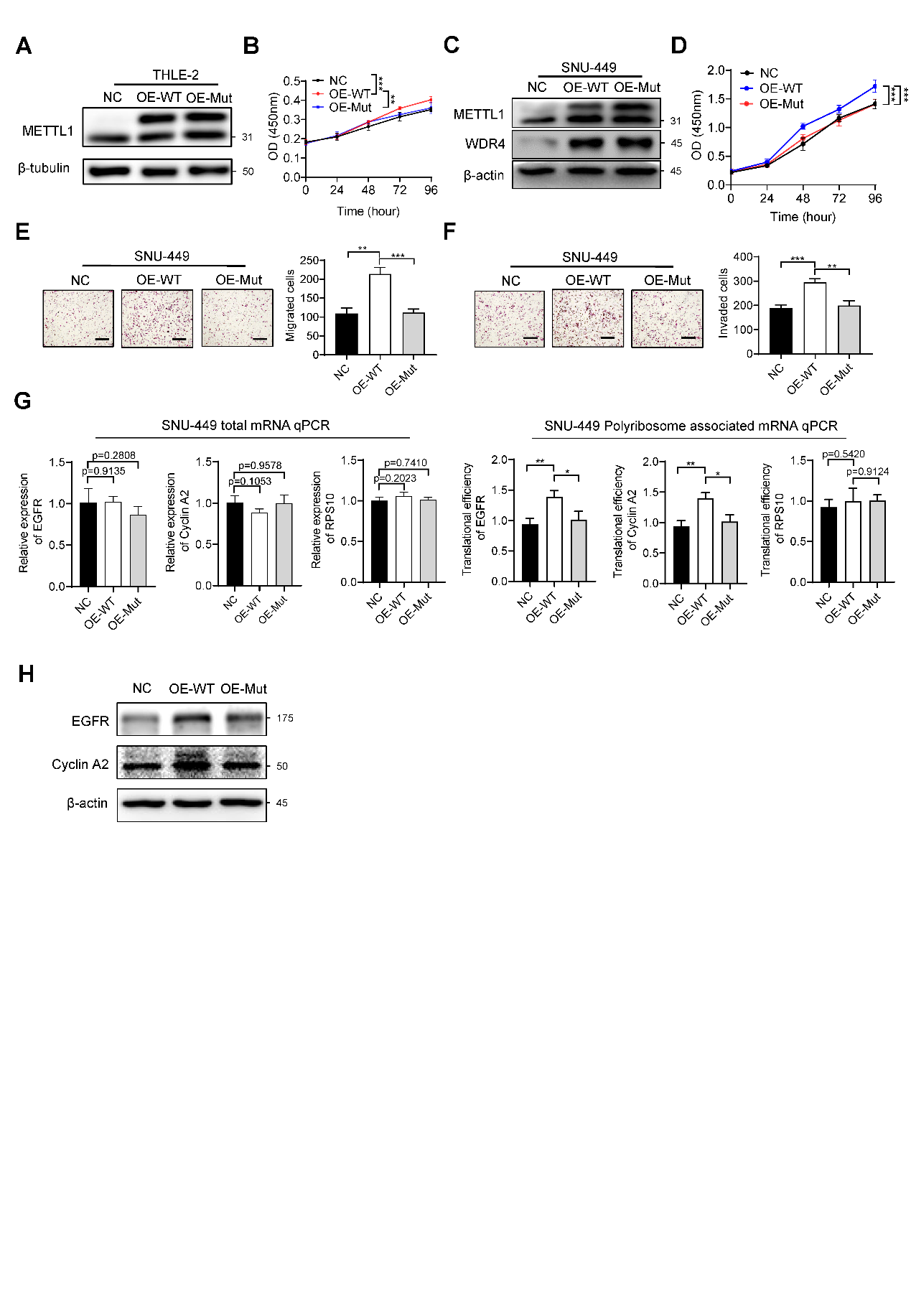
**

**Figure S13. Overexpression of METTL1 promotes HCC cells progression and translation of Cyclin A2 and EGFR. (A)** Confirmation of METTL1 overexpression by western blot in THLE-2 cells with overexpression of wild type or mutant METTL1. **(B)** CCK-8 assay of THLE-2 cells with overexpression of wild type or mutant METTL1. Data presented as mean ± SD (Six technical replicates). **(C)** Confirmation of METTL1 overexpression by western blot in SNU-449 cells with overexpression of wild type or mutant METTL1. **(D)** CCK-8 assay of SNU-449 cells with overexpression of wild type or mutant METTL1. Data presented as mean ± SD (Six technical replicates). **(E)** Representative images and quantification of migration in SNU-449 cells with overexpression of wild type or mutant METTL1. Scale bar, 500 μm. Data presented as mean ± SD (Three technical replicates). **(F)** Representative images and quantification of invasion in SNU-449 cells with overexpression of wild type or mutant METTL1. Scale bar, 500 μm. Data presented as mean ± SD (Three technical replicates). **(G)** Relative expression and translation efficiency of Cyclin A2 and EGFR mRNA in SNU-449 cells with overexpression of wild type or mutant METTL1. **(H)** Western blot of Cyclin A2 and EGFR in SNU-449 cells with overexpression of wild type or mutant METTL1. Data presented as mean ± SD. *p <0.05, **p < 0.01, ***p < 0.001 by Student’s t test or the Mann-Whitney U test. All the in vitro assays were biologically repeated for 3 times. NC, negative control; OE-WT, overexpression of wild type METTL1; OE-Mut, overexpression of mutant METTL1.

**
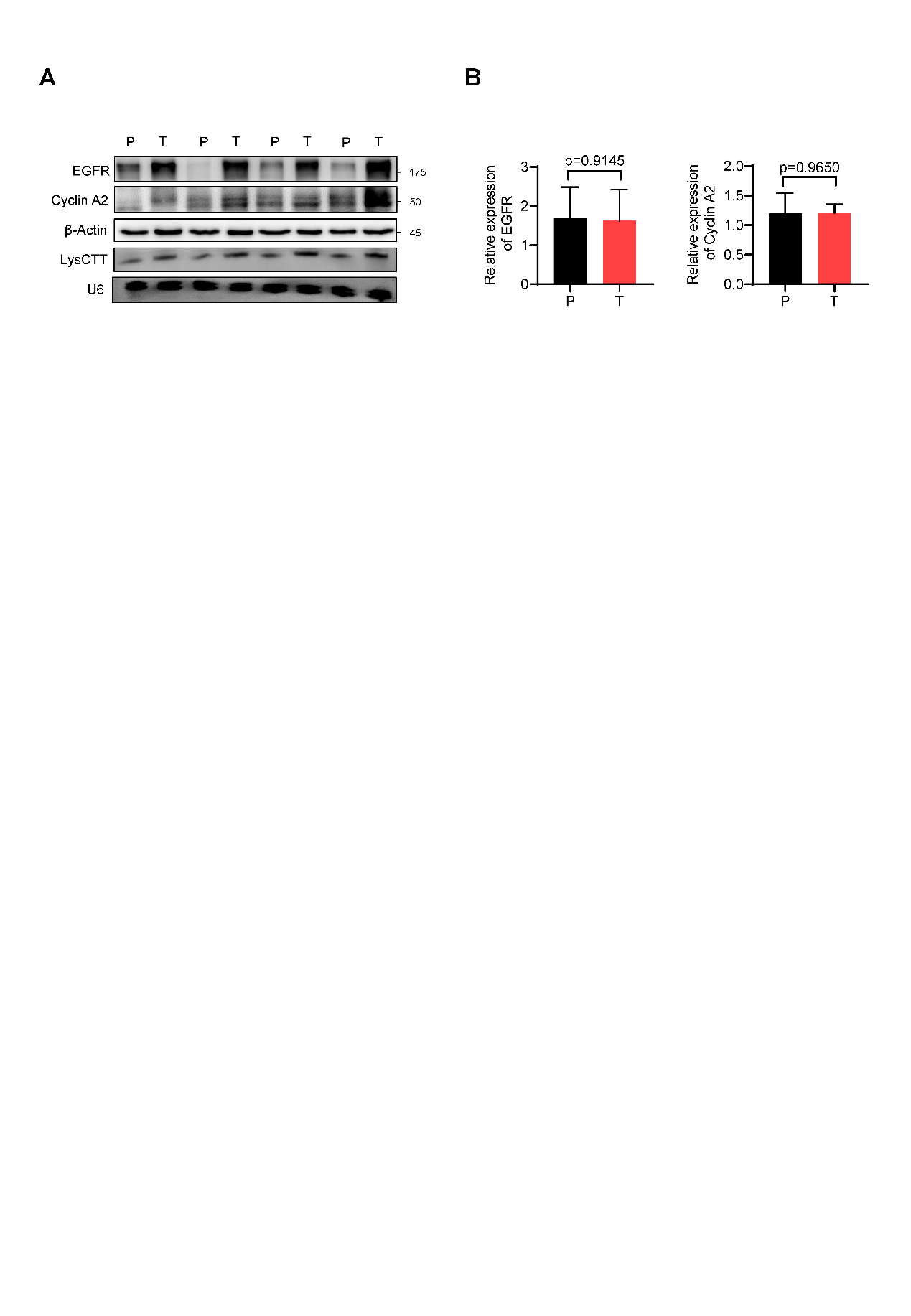
**

**Figure S14. Expression levels of EGFR, Cyclin A2 and tRNA-LysCTT in HCC clinical samples. (A)** Expression levels of EGFR, Cyclin A2 and tRNA-LysCTT in in four pairs of HCC tissues and corresponding peri-tumor tissues. **(B)** qRT-PCR analysis of EGFR and Cyclin A2 in four pairs of HCC tissues and corresponding peri-tumor tissues. Data presented as mean ± SD (n=4). *p <0.05, **p < 0.01, ***p < 0.001 by Student’s t test, one-way ANOVA or the Mann-Whitney U test unless specified. HCC, hepatocellular carcinoma; P, peri-tumor tissue; T, tumor tissue.
